# A pilot randomized controlled trial of an online intervention for Hodgkin lymphoma survivors to increase knowledge about late effects and recommended screening

**DOI:** 10.1007/s11764-024-01587-2

**Published:** 2024-04-20

**Authors:** Tara J. Rick, Smitha Sagaram, Patricia I. Jewett, Hee Yun Lee, Karim T. Sadak, Lucie M. Turcotte, Rachel I. Vogel, Anne Blaes

**Affiliations:** 1 Department of Medicine, Division of Hematology/Oncology, University of Minnesota, Minneapolis, USA; 2 University of Minnesota Masonic Cancer Center, Minneapolis, USA; 3 Banner MD Anderson Cancer Center, Phoenix, AZ, USA; 4 School of Social Work, University of Alabama, Tuscaloosa, USA; 5 Department of Pediatrics, Division of Pediatric Hematology and Oncology, University of Minnesota, Minneapolis, USA; 6 Department of Obstetrics, Gynecology and Women’s Health, University of Minnesota, Minneapolis, USA

**Keywords:** Breast cancer, Cancer screening, Chest radiation, Hodgkin lymphoma, Secondary cancer, Survivor, Online intervention

## Abstract

**Background:**

Hodgkin lymphoma (HL) survivors who received chest radiotherapy are at risk for breast cancer and cardiovascular disease, but screening adherence is low. We assessed the acceptability/feasibility of a web-based educational intervention and its impact on knowledge of health risks and screening.

**Methods:**

HL survivors were randomized to either an interactive online educational intervention or handouts only. Surveys were completed at baseline and 3 months post-intervention. We described the acceptability/feasibility of the intervention and compared knowledge between groups.

**Results:**

Fifty-two HL survivors participated; 27 in the intervention group and 25 in the control group. Eighteen (66%) intervention participants completed the intervention and reported high acceptability (89–100%). At baseline, adherence to breast cancer screening was low across all participants. Post-intervention, those in the intervention group more often than controls correctly identified breast cancer and echocardiogram screening guidelines (35% vs. 28%, *P* = 0.02 and 82% vs. 52%, *P* = 0.04) and reported knowing how to address potential complications from cancer treatments (87% vs. 64%, *P* = 0.03). We detected no increase in screening behavior post-intervention.

**Conclusion:**

Online education modules for high-risk HL survivors are an acceptable method to improve knowledge of health risks and screening guidelines. Future interventions should focus on improving screening uptake in this population.

**Implications for Cancer Survivors:**

Web-based learning can be useful in increasing cancer survivor knowledge of their unique risks and screening recommendations but does not necessarily change patient behavior. Involvement in a cancer survivorship program can help assess individual barriers and monitor uptake of screening.

## Introduction

Hodgkin lymphoma (HL) affects approximately 9000 young adults and children annually in the United States, with cure rates exceeding 80% [1, 2]. Although mortality rates for HL have decreased over the last several decades, survivors are at risk for serious late effects from their cancer treatments, most notably secondary malignancies and cardiovascular disease [3, 4]. Breast cancer accounts for more than 40% of the excess risk for a second cancer among female HL survivors despite reductions in radiation exposure over time [5–7]. The risk of cardiovascular disease approaches 17% by age 35 years among HL survivors, and the most common cardiovascular diagnoses include coronary artery disease, valvular heart disease, cardiomyopathy, and heart failure [8]. At the age of 54 years, HL survivors had a mortality rate similar to that of a 71-year-old in the general population [6]. Therefore, lifesaving treatment in HL survivors may also shorten one’s life span [7].

Many late complications of HL can be mitigated if detected and treated early. Comprehensive, evidence-based guidelines from the National Comprehensive Cancer Network (NCCN) and the Children’s Oncology Group (COG) are commonly used to guide screening practices [9, 10]. However, adherence to breast cancer and cardiovascular screening guidelines remains low among HL survivors [11–13]. Reasons for this are multifactorial; primary care providers often lack knowledge about appropriate screening practices for high-risk cancer survivors, such that some HL survivors do not receive appropriate long-term follow-up referrals and testing [14]. In addition, many HL survivors may be unaware of their treatment-related health risks, and there may also be financial and systemic barriers to accessing these surveillance studies [13, 15].

Educational interventions could help increase the number of HL survivors who receive recommended preventative surveillance to mitigate breast cancer and cardiovascular risk. Not all HL patients are aware of the need for long-term follow-up, and few interventions have aimed at increasing post-HL screening compliance [16]. For example, previous interventions that used mailed materials or telephone counseling were effective in increasing mammography screening rates among high-risk cancer survivors, however, they did not increase the rate of breast MRI [17, 18]. Over time, patients have increasingly become more comfortable getting health information from the internet, moving away from reliance on printed materials [19]. Web-based interventions have been effective in modifying health behaviors in cancer survivorship care, such as diet, physical activity, and weight management [20–30]. However, there is less data focusing on web-based interventions for Hodgkin lymphoma survivors, such as education of late effects and recommended screenings.

We developed and pilot-tested an online intervention, Mobile Application for Survivorship Care (MAPS), tailored for HL survivors to educate them about the late effects of treatment and recommended screening. The primary goal was to determine the feasibility and acceptability of MAPS to deliver survivorship care recommendations in this high-risk group. Secondary outcomes included (a) increasing HL survivors’ knowledge of their long-term health risks and recommended preventive care, with a focus on breast cancer and adverse cardiovascular outcomes, and (b) increasing uptake of recommended preventive health screenings using MAPS mobile application compared to printed material.

## Methods

### Study design and population

We conducted a pilot randomized controlled trial of the MAPS intervention, described in more detail below, among 52 female HL survivors between 2019 and 2020. Females diagnosed with HL before 2011, treated with mantle or chest radiation, and with an attained age of at least 18 years at the time of recruitment were eligible. Participants were required to be English-speaking, mentally/physically competent to complete a questionnaire and provide consent, and willing to learn mobile device technology. HL survivors who were hearing or visually impaired in a way that would prevent them from using a mobile application and those who were diagnosed with breast cancer or were previously seen in the cancer survivorship clinic at the University of Minnesota where they would have already received counseling and specific recommendations on screening were excluded from participation. This study was approved by the University of Minnesota Institutional Review Board (IRB #: 1610S97201), and all participants provided written informed consent prior to study participation.

### Recruitment

Potentially eligible participants were identified based on a review of electronic medical records and a database of cancer survivors at the University of Minnesota. Initial invitation letters were mailed and followed by a telephone call from the study coordinator to assess interest and confirm eligibility. For those not interested (active decline) or unresponsive to the invitation and two subsequent outreach attempts (passive decline), no further contact was made. Following written consent and completion of the baseline survey, participants were randomized 1:1 to the intervention or control arm. SAS 9.4 was used to generate random sequence numbers based on block randomization. The allocations were placed in opaque, sealed envelopes and opened sequentially by the study coordinator to randomize participants.

### Intervention

The app modules were developed and tested by a multidisciplinary team based on the Fogg Behavioral Model of Mobile Persuasion [31], and using input from focus groups of HL survivors. Focus groups were conducted in June 2017 among 10 HL survivors (1) with secondary complications of breast cancer or cardiovascular disease or (2) at risk for secondary complications. During the focus groups, patient experience, awareness of potential late effects and screening, barriers to screening, and preferences for receiving information were assessed. Key findings included (1) a general awareness that additional screening may be indicated with a gap in knowledge on what to do and (2) that most preferred to receive information about recommended health screening digitally. Potential barriers to screening included conflicting information, low confidence that they are receiving the correct information, financial concerns/lack of insurance coverage, or avoidance/rejection of information (i.e., “I feel good so why should I do additional testing?”).

**Fig. 1:**
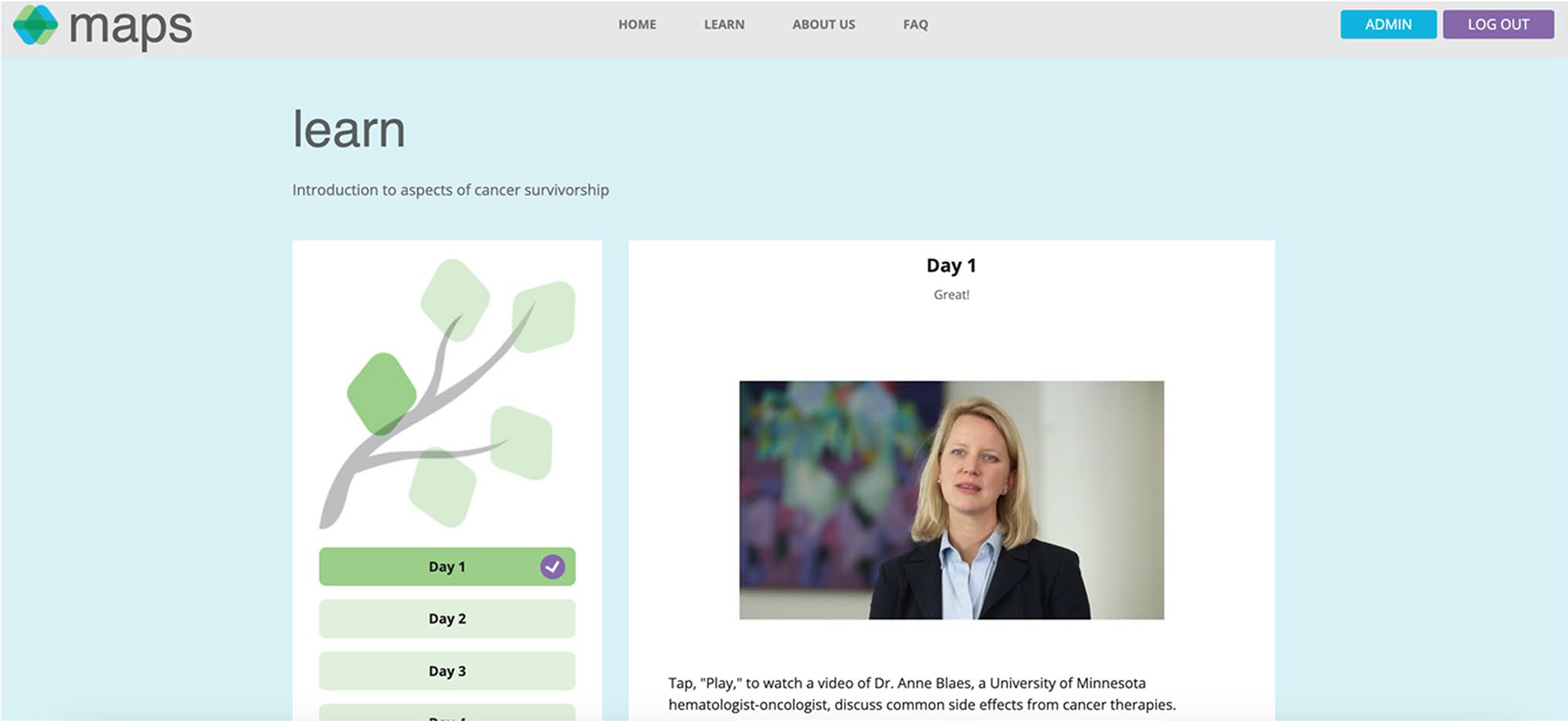
Screenshot of Mobile Applications for Survivorship Care (MAPS) online learning module

Five educational online modules were created, incorporating the focus group findings, and utilizing videos and graphics to enhance messaging (Fig. 1). These tailored and interactive modules were provided to participants in the intervention group, spread out over 5 days. Each daily module took approximately 5 min to complete. The modules prompted participants to interact and respond to questions, but the educational information was delivered to all participants. Prompted daily topics included (1) the importance of cancer survivorship care plans, (2) treatment-related cardiovascular complications and secondary cancers, (3) individual risk factors based on prior therapies, (4) screening guidelines, and (5) communication strategies, healthy lifestyle, selfcare, and follow-up. Participant access to MAPS was password protected. Study coordinators were able to monitor if the modules were completed in entirety or not with website metrics; but for those with only partial completion, we did not capture the percent completed nor which modules were partially vs fully completed.

Participants in the control group electronically received a 5-page handout tailored to the study population and an educational handout from the American Society of Clinical Oncology (ASCO) for cancer survivors, i.e., these handouts described referenced published literature around survivorship care plans, second cancer, and cardiovascular screening, and information regarding healthy lifestyle following cancer treatment [32]. The handouts included the same topics as the intervention group. At the completion of the study, participants in the control group were given access to the online modules.

### Study procedures

After providing written consent, study participants were asked to complete an online baseline survey via REDCap electronic data capture tool hosted at the University of Minnesota. Following completion of the baseline survey and randomization, those randomized to the intervention group were instructed to download the MAPS application and view all materials. Both groups were electronically sent the educational handouts. A final REDCap survey was sent to all participants 3 months after baseline.

### Study measures

The surveys were adapted from previously developed and utilized surveys in previous studies, results of focus group, and with collaboration with the Office of Measurement Services at the University of Minnesota [33–35]. The primary outcome was usability, defined as the feasibility, measured by completion rate (the proportion of individuals randomized to the intervention group who completed all study-related procedures), and acceptability, measured by satisfaction with the app-based intervention. Six items were developed for the study to identify satisfaction with attributes of the intervention, similar to questions we had previously used to assess a previous mobile app intervention (Table 2) [36]. Acceptability of printed materials was not measured in the control group. We were interested in several secondary outcomes: (1) HL survivors’ knowledge of risk of secondary cancers, adverse cardiovascular outcomes, and of screening guidelines. This knowledge was assessed both at baseline and in the post-intervention survey. Questionnaire items are depicted in Table 3. (2) Adherence to recommended screening guidelines (mammogram alone, mammogram and breast MRI or MRI alone) or intention to screen. Additionally, self-reported demographic and clinical information such as age, education, rural/urban location defined by ZIP-code-based Rural Urban Commuting Area (RUCA) codes, and annual household income were collected. Cancer- and treatment characteristics including age at diagnosis and treatments received for HL (surgery, chemotherapy, and radiation) were abstracted from medical records.

### Statistical considerations

The planned sample size of 60 participants for this pilot study was primarily a function of feasibility and to provide preliminary estimates of the acceptability and effectiveness of the intervention. Based on previous experience, we anticipated approximately 60% of those recruited to the study would complete the 3-month follow-up survey, and therefore a final sample size of 34 participants with complete data was expected. For analysis of knowledge scores, a sample size of 34 (17 in each arm) achieves 80% power to detect an effect size of 1.0 using a two-sided, two-sample *t*-test with a significance level of 0.05. Recruitment was stopped for this study at 52 participants due to significant disruptions to research implementation due to the COVID-19 pandemic.

Descriptive analyses were conducted to compare demographic and clinical characteristics between the randomized groups. Frequencies of correct answers to the knowledge questions regarding potential complications from prior HL treatment and breast cancer and cardiovascular screening guidelines were compared between the intervention and control groups at baseline and post-intervention using Chi-squared and Fisher’s exact tests. The primary analysis was intention-to-treat, analyzing participants in the group they were randomized to regardless of intervention engagement. A secondary analysis was completed, including only those who completed the MAPS learning modules in the intervention group. SAS 9.4 was used for all statistical analyses, and *P* values < 0.05 were considered statistically significant.

## Results

### Primary outcome-feasibility and acceptability

**Fig. 2:**
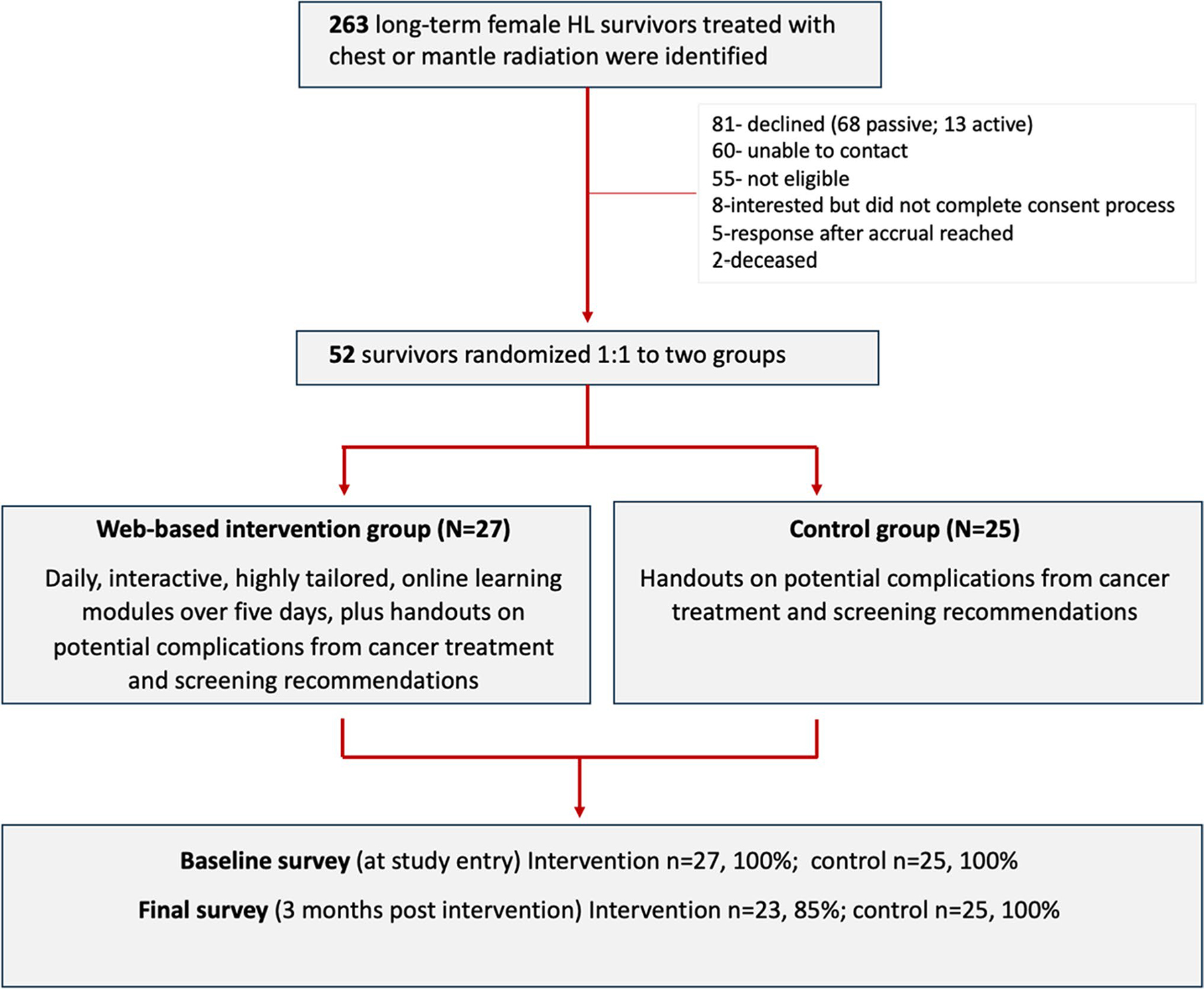
Patient selection and randomization, Mobile Applications for Survivorship Care (MAPS) Study, 2020

A total of 263 potentially eligible participants were invited. A total of 52 (23%) consented to participate; 27 in the intervention group and 25 in the control group. All 25 individuals in the non-intervention group completed the follow-up survey. Out of the 27 individuals in the intervention group, four individuals did not complete the follow-up survey (Fig. 2).

Baseline characteristics did not differ significantly between the intervention and the control group either (Table 1). The median age at baseline was 48.1 (range 28.0–71.8) years. The median time since diagnosis was 21.3 (range 7.8–48.5) years. Most participants were white (98.1%) and urban (90.4%). All had health insurance, most had at least a college degree (65.4%), and 46.2% had an annual household income of $100,000 or higher. About half (51.9%) had received surgery, and 86.5% had received chemotherapy. Self-reported adherence to annual mammography and breast MRI screening was low (53.9% and 6.1%, respectively).

When comparing the baseline characteristics of the four participants who did not complete the follow-up survey with the baseline characteristics of the 23 follow-up completers in the intervention group, there were no significant differences, and the same was true when comparing these four individuals with all 48 follow-up completers regardless of randomization status. Further, when comparing the baseline characteristics of participants randomized to the intervention who did (*N* = 18) versus did not complete all the online modules (*n* = 9), we found no significant differences. Given the small sample size of non-completers, it is possible that limited power kept us from capturing some differences.

Over half (66.7%) of the participants randomized to the intervention completed all five app modules. Among the 18 participants in the intervention group who reported the modules, all indicated that they would recommend the learning modules to other HL survivors (feasibility; Table 2). Nearly all the 18 participants who completed the intervention in entirety reported being very or somewhat satisfied with the information presented (acceptability), including relevance (100%), learning about cancer survivorship (88.9%), tone of messages (100%), level of interaction (100%), length of daily messaging (94.4%), and overall website/learning (94.4%).

### Secondary outcomes-knowledge and screening

At baseline, participants in both the intervention and control groups were similar regarding their knowledge of treatment complications and screening guidelines (Table 3): 81.5% of participants in the intervention group and 76.0% of controls reported awareness of potential complications from previous HL treatments. However, only 33.3% of participants in the intervention group and 32.0% of controls reported knowing what they could do to reduce their risk of complications from prior treatments, and only 25.9% of participants in the intervention group and 28.0% of controls correctly identified national guidelines on breast cancer screening for HL survivors treated with chest radiation. Sixty-three percent of participants in the intervention group and 52.0% of controls reported that they had been advised to receive preventive health screenings as high-risk HL survivors. Only 14.8% of participants in the intervention group and 16.0% of controls reported that they had received a written summary of their diagnosis and treatments received. Most (76.4%) participants in the intervention group and control (72.0%) groups reported that they had never attended a clinic for the purpose of late effects follow-up. None of these baseline items differed significantly between the intervention and the control group.

At 3 months post-intervention (Table 3), individuals assigned to the intervention group were more likely than controls to report knowing what they could do to reduce the risk of secondary complications from cancer treatments (intention to treat analysis: 87.0% vs. 64.0%, *P* = 0.07; treatment received analysis: 94.4% vs. 64.0%, *P* = 0.03). The intervention group more often correctly identified national guidelines on breast cancer screening (intention to treat analysis: 34.8% vs. 28.0%, *P* = 0.02; treatment received analysis: 38.9% vs. 28.0%, *P* = 0.01), as well as recommendations for screening echocardiograms (intention to treat analysis: 81.8% vs. 52.0%, *P* = 0.10; treatment received analysis: 88.2% vs. 52.0%, *P* = 0.04). No statistically significant differences were identified, either in the intention-to-treat or in the treatment-received analyses, between the control and intervention groups regarding scheduling or completing a mammogram, breast MRI or other screening appointments, or intention to screen post-intervention (Table 4). However, there was a tentatively higher proportion among individuals in the intervention group intending to complete a breast MRI (17.0% vs. 0.0%, *P* = 0.08).

## Discussion

Our findings contribute to a growing body of evidence that tailored, interactive, web-based resources are feasible and highly acceptable in delivering education on cancer survivorship care [37, 38]. Web-based interventions have the protentional to reach patients that are not engaged in a formal cancer survivorship program and bring awareness to interventions that influence mortality such as screening for late effects and adopting a healthy lifestyle [39, 40]. Also, the interactive modules may be better at engaging patients who prefer to learn with in this manner versus reading educational material. In addition, findings are potentially applicable to other cancer survivorship populations, e.g., adolescent, and young adult (AYA) populations, targeting a variety of cancer diagnoses, as this population is increasingly relying on web-based resources in various aspects of their lives. One challenge is designing a resource that is at the appropriate educational level for a great majority of patients. In this study, participants were highly educated, and according to feedback, some did not complete the modules because they felt it was too basic. When comparing (data not shown) baseline answers to the knowledge questions and education levels among those in the intervention group who did and did not complete the modules, we found no significant differences.

Consistent with prior research, most HL survivors in our study had not received a cancer survivorship care plan and most had not received recommended breast cancer and cardiovascular screenings [41]. At the 3-month follow-up, knowledge about screening guidelines was higher among those randomized to the intervention group compared with controls; however, no significant differences were observed in screening behaviors or intention to screen. More individuals from the intervention group reported intention to schedule a breast MRI at 3-month follow-up although this did not achieve statistical significance. Reasons for the lack of impact on screening behaviors are likely multifactorial. The end of our study coincided with the early COVID-19 pandemic. It is likely that scheduling screening tests was difficult or significantly delayed during the early phase of the pandemic when many cancer services deemed non-essential were canceled or postponed. Other potential reasons for not scheduling breast cancer screening may be lack of insurance coverage and cost (particularly for breast MRI), conflicting recommendations from primary care providers, logistics around transportation and time from work for cancer screening, and anxiety around screening (scan anxiety or claustrophobia with MRI), and perhaps the “ostrich problem” (delaying screening to avoid potentially confronting results they would mentally prefer to avoid [12, 42, 43]. Future work under post-pandemic circumstances is needed to assess whether long-term screening behaviors change after similar web-based educational interventions for HL survivors.

Limitations of this study include a small sample size, which was by design for this pilot study and thus was underpowered for the analyses of secondary endpoints. Data was drawn from a single Midwest academic institution. Most participants were highly educated, insured, non-Hispanic White females, which potentially limits generalizability to more diverse populations. We were unable to evaluate partial module completion to determine which modules had the highest or lowest engagement indicating if certain modules may be more useful. Importantly, this study was conducted in part during the early COVID-19 pandemic, which likely affected screening uptake following the intervention.

## Conclusion

This study provides evidence that a tailored online intervention is a feasible, acceptable, and potentially effective way to provide survivorship education for Hodgkin lymphoma survivors. Further research is needed to investigate ways to further improve engagement with the app and understand barriers in the uptake of screening tests for late effects in HL survivors.

## Supplementary Material

Supplementary file2

Supplementary file1

## Figures and Tables

**Table 1:** Participant characteristics at baseline, *N* = 52, Mobile Applications for Survivorship Care (MAPS) Study, 2020

Characteristic	Everyone (*N* = 52)		Control (*N* = 25)		Intervention (*N* = 27)		*P*
	*N*	Median (Range)	*N*	Median (Range)	*N*	Median (Range)	

Age at survey, years	52	48.1 (28.0–71.8)	25	47.4 (28.0–68.1)	27	50.0 (35.8–71.8)	0.24
Age at diagnosis, years	48	25.0 (9.0–53.2)	23	25.1 (9.0–48.0)	25	24.9 (14.7–53.2)	0.71
Time since HL diagnosis, years	48	21.3 (7.8–48.5)	23	17.2 (7.8–43.7)	25	23.2 (10.9–48.5)	0.20
	***N***	%	***N***	%	***N***	%	
Race/ethnicity							> 0.99
White	51	98.1	25	100.0	26	96.3	
Not reported	1	1.9	0	0.0	1	3.7	
Urbanicity							0.18
Rural	5	9.6	4	16.0	1	3.7	
Urban	47	90.4	21	84.0	26	96.3	
Insurance							NA
Insured	51	100	25	100.0	27	100.0	
Education							0.84
At least college degree	34	65.4	16	64.0	18	66.7	
No college degree	18	34.6	9	36.0	9	33.3	
Annual Income							0.46
< $50,000	3	5.8	2	8.0	1	3.7	
$50,000–99,999	16	30.8	6	24.0	10	37.0	
≥ $100,000	24	46.2	14	56.0	10	37.0	
Prefer not to say	9	17.3	3	12.0	6	22.2	
HL treatments received							0.26
Surgery							
No	25	48.1	10	40.0	15	55.6	
Yes	27	51.9	15	60.0	12	44.4	
Chemotherapy							0.70
No	7	13.5	4	16.0	3	11.1	
Yes	45	86.5	21	84.0	24	88.9	
Screening behavior at baseline							0.39
At least annual mammogram							
No	24	46.2	10	40.0	14	51.9	
Yes	28	53.9	15	60.0	13	48.2	
At least annual breast MRI							> 0.99
No	46	93.9	23	95.8	23	92.0	
Yes	3	6.1	1	4.2	2	8.0	

**Table 2:** Acceptability and feasibility of the web-based intervention in participants who completed the modules, *N* = 18, Mobile Applications for Survivorship Care (MAPS) Study, 2020

Questionnaire item	*N*	%

Relevancy of information		
Very satisfied	14	77.8
Somewhat satisfied	4	22.2
Somewhat dissatisfied	0	0.0
Very dissatisfied	0	0.0
What I learned about cancer survivorship after Hodgkin’s lymphoma		
Very satisfied	12	66.7
Somewhat satisfied	4	22.2
Somewhat dissatisfied	2	11.1
Very dissatisfied	0	0.0
Tone of messages		
Very satisfied	17	94.4
Somewhat satisfied	1	5.6
Somewhat dissatisfied	0	0.0
Very dissatisfied	0	0.0
Level of interaction (videos, questions, quiz, etc.)		
Very satisfied	15	83.3
Somewhat satisfied	3	16.7
Somewhat dissatisfied	0	0.0
Very dissatisfied	0	0.0
Length of daily messaging		
Very satisfied	15	83.3
Somewhat satisfied	2	11.1
Somewhat dissatisfied	1	5.6
Very dissatisfied	0	0.0
Overall satisfaction with the website/learning modules		
Very satisfied	12	66.7
Somewhat satisfied	5	27.8
Somewhat dissatisfied	1	5.6
Very dissatisfied	0	0.0
Would you recommend the online learning modules to other individuals who have had Hodgkin’s lymphoma?		
No	0	0.0
Yes	18	100
Would you share the learning modules with family or friends?		
No	6	33.3
Yes	12	66.7

**Table 3:** Knowledge questions at baseline vs. post-intervention (3-month follow-up), *N* = 52, Mobile Applications for Survivorship Care (MAPS) Study, 2020

Knowledge question	Baseline					Post-intervention							
	Controls		Intervention		*P*	Controls		Intention to treat (*N* = 23)		*P*	Treatment received (*N* = 18)		*P*
								
	*N*	*%*	*N*	*%*		*N*	*%*	*N*	*%*		*N*	*%*	

Do you feel the previous treatments you received could cause serious future health problems?					0.62					0.38			0.40
No	3	12.0	1	3.9		3	12.0	3	13.0		3	16.7	
Yes*	19	76.0	22	81.5		19	76.0	20	87.0		15	83.3	
I do not know	3	12.0	4	14.8		3	12.0	0	0.0		0	0.0	
Do you know what you can do to reduce your risk for certain kinds of complications from your prior cancer treatments?					0.92					0.07			0.03
No	17	68.0	18	66.7		9	36.0	3	13.0		1	5.6	
Yes*	8	32.0	9	33.3		16	64.0	20	87.0		17	94.4	
Have you been advised to receive different health screenings for prevention based on your prior health problems?					0.67					0.61			0.46
No	10	40	9	33.3		5	20.0	6	26.1		5	27.8	
Yes*	13	52	17	63.0		20	80.0	16	69.6		12	66.7	
I don’t know	2	8	1	3.7		0	0.0	1	4.4		1	5.8	
Have you ever attended a clinic for the purpose of late effects follow-up?					0.29					0.21			0.40
No	18	72.0	21	76.4		18	72.0	12	52.2		10	55.6	
Yes	7	28.0	4	14.8		7	28.0	9	39.1		7	38.9	
I do not know	0	0.0	2	7.4		0	0.0	2	8.7		1	5.6	
Have you ever received a written summary of your prior cancer and treatment?					0.82					0.47			0.37
No	19	76	19	70.4		14	56.0	16	69.6		14	77.8	
Yes	4	16	4	14.8		7	28.0	6	26.1		3	16.7	
I do not know	2	8	4	14.8		4	16.0	1	4.4		1	5.6	
The current national breast cancer screening guidelines for Hodgkin lymphoma survivors who have had chest radiation are the following:					0.57					0.02			0.01
Breast MRI and mammogram beginning at age 25 years or 8 years after therapy*	7	28.0	7	25.9		7	28.0	8	34.8		7	38.9	
Mammograms alone at age 50 years	1	4.0	1	3.7		0	0.0	1	4.4		0	0.0	
Mammograms alone at age 40 years	4	16.0	1	3.7		6	24.0	0	0.0		0	0.0	
Breast MRI and Mammogram beginning at age 40 years	1	4.0	3	11.1		4	16.0	10	43.5		9	50.0	
I do not know	12	48.0	15	55.6		8	32.0	4	17.4		2	11.1	
Hodgkin lymphoma survivors who have received chest radiation have a risk of cardiovascular disease equivalent to the general population					0.66					0.10			0.03
True	7	28.0	5	18.5		6	24.0	7	30.4		4	22.2	
False*	6	24.0	6	22.2		12	48.0	15	65.2		14	78.8	
I do not know	12	48.0	16	59.3		7	28.0	1	4.4		0	0.0	
Knowing that you are at increased risk for complications from your prior cancer treatment will help you take extra steps to prevent or find future cancers earlier					0.80					0.80			0.75
True	23	92.0	25	92.6		23	92.0	21	91.3		16	88.9	
False	1	4.0	0	0.0		2	8.0	1	4.4		1	5.6	
I do not know	1	4.0	2	7.4		0	0.0	1	4.4		1	5.6	
Monitoring cholesterol, blood pressure and avoiding tobacco use can help minimize cardiovascular complications after cancer therapy					0.83					0.48			NA
True*	21	84.0	23	88.5		25	100.0	18	100.0		18	100.0	
False	1	4.0	0	0.0		0	0.0	0	0.0		0	0.0	
I do not know	3	12.0	3	11.5		0	0.0	0	0.0		0	0.0	
Hodgkin lymphoma survivors who have had chemotherapy and chest radiation should have received at least one screening echocardiogram after completing their cancer therapy					0.89					0.07			0.04
True*	11	44.0	10	37.0		13	52.0	18	81.8		15	88.2	
False	1	4.0	1	3.7		2	8.0	1	4.6		0	0.0	
I do not know	13	52.0	16	59.3		10	40.0	3	13.6		2	11.8	

* Considered a correct answer for knowledge of risk and of screening recommendation

**Table 4:** Scheduling screening post-intervention, *N* = 52, Mobile Applications for Survivorship Care (MAPS) Study, 2020

	Controls (*N* = 25)		Intervention, intention-to-treat analysis, (*N* = 23)			Intervention, treatment-received analysis (*N* = 18)		
	*N*	%	*N*	%	*P*	*N*	%	*P*

In the past 3 months, have you received or scheduled a mammogram?					> 0.99			> 0.99
No	13	52.0	12	52.2		10	55.6	
Yes, I received a mammogram	3	12.0	3	13.0		2	11.1	
I have scheduled a mammogram but have not had the appointment yet	3	12.0	2	8.7		2	11.1	
No, but I am planning to schedule a mammogram soon In the past 3 months, have you received or scheduled a breast MRI?	6	24.0	6	26.1	0.08	4	22.2	0.07
No	24	96.0	18	78.3		14	77.8	
Yes	0	0.0	0	0.0		0	0.0	
I have scheduled a breast MRI but have not had the appointment yet	1	4.0	1	4.4		1	5.6	
No, but I am planning to schedule a breast MRI soon In the past 3 months, have you received or scheduled any other screening appointments?	0	0.0	4	17.4	0.61	3	16.7	0.40
No	17	68.0	14	60.9		10	55.6	
Yes, I have received/scheduled or am planning to schedule a different screening appointment (please provide details below)	8	32.0	9	39.1		8	44.4	
