# Supplementary material for: A pilot randomized controlled trial of an online intervention for Hodgkin lymphoma survivors to increase knowledge about late effects and recommended screening
Source: J Cancer Surviv. 2024 Apr 20;19(6):1781–92. doi: 10.1007/s11764-024-01587-2 (PMC11490585; doi:10.1007/s11764-024-01587-2)
Supplement: Supplementary file 1 — Supplementary file1 (PDF 3254 KB) [file 11764_2024_1587_MOESM1_ESM.pdf]

ASCO® answers

Cancer Survivorship

Trusted Information to Help Manage Your Care from the American Society of Clinical Oncology

Cancer.Net
Doctor-Approved Patient Information from ASCO®

ASCO® Cancer.Net

ABOUT ASCO

Founded in 1964, the American Society of Clinical Oncology, Inc. (ASCO®) is committed to making a world of difference in cancer care. As the world’s leading organization of its kind, ASCO represents nearly 45,000 oncology professionals who care for people living with cancer. Through research, education, and promotion of the highest-quality patient care, ASCO works to conquer cancer and create a world where cancer is prevented or cured, and every survivor is healthy.

ASCO furthers its mission through Cancer.Net and its Conquer Cancer Foundation.

Cancer.Net (www.cancer.net) brings the expertise and resources of ASCO to people living with cancer and those who care for and about them. All the information and content on Cancer.Net is developed and approved by members of ASCO, making Cancer.Net an up-to-date and trusted resource for cancer information.

Conquer Cancer (www.conquer.org) funds research into every facet of cancer to benefit every patient, everywhere. Conquer Cancer helps turn science into a sigh of relief for patients around the world by supporting groundbreaking research and education across cancer’s full continuum.

Learn more at www.ASCO.org. Follow us on Facebook, Twitter, LinkedIn, and YouTube.

MILLIONS OF PEOPLE RELY ON CANCER.NET FOR:

- Information on 120+ cancer types
- Information on navigating cancer care
- Coping and survivorship resources

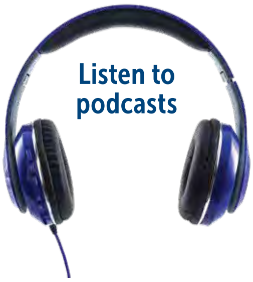

GET YOUR CANCER INFORMATION IN THE FORMAT YOU WANT:

Download the app

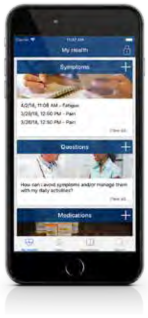

Watch videos

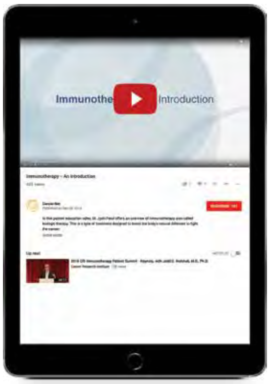

Browse the website

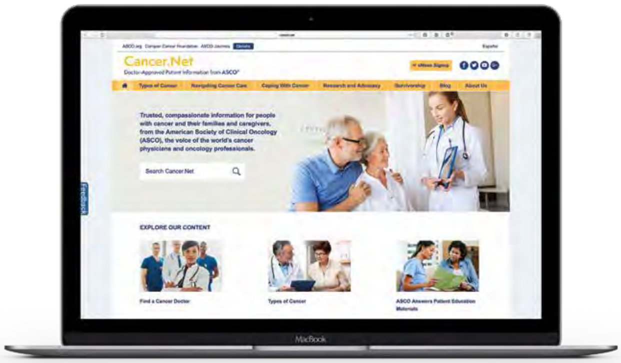

ASCO patient education programs are supported by:

CONQUER CANCER®
THE ASCO FOUNDATION

Cancer Survivorship

Table of Contents

Introduction 2

My Health Care Team 3

What Is Survivorship? 4

Defining survivorship 4

Follow-Up Care 6

Watching for recurrence 6

Managing late and long-term side effects 8

Keeping a personal health record 11

Treatment summary and survivorship care plan 12

Life After Treatment: What to Expect 14

Fear of recurrence 14

A new perspective on your health 16

Personal reflection 18

Changes within families and relationships 19

Starting or expanding your family 21

Going back to work 24

Managing your finances 27

Support for Coping With Challenges 29

Cancer rehabilitation 29

Survivorship support groups 34

Other support resources 35

Making a Difference 36

Survivorship Dictionary 38

ASCO ANSWERS is a collection of oncologist-approved patient education materials developed by ASCO for people with cancer and their caregivers.

The ideas and opinions expressed in *ASCO Answers: Cancer Survivorship* do not necessarily reflect the opinions of ASCO or the Conquer Cancer Foundation of ASCO. The information in this guide is not intended as medical or legal advice, or as a substitute for consultation with a physician or other licensed health care provider. Patients with health-related questions should call or see their physician or other health care provider promptly, and should not disregard professional medical advice, or delay seeking it, because of information encountered in this guide. The mention of any product, service, or treatment in this guide should not be construed as an ASCO endorsement. ASCO is not responsible for any injury or damage to persons or property arising out of or related to any use of ASCO's patient education materials, or to any errors or omissions.

Introduction

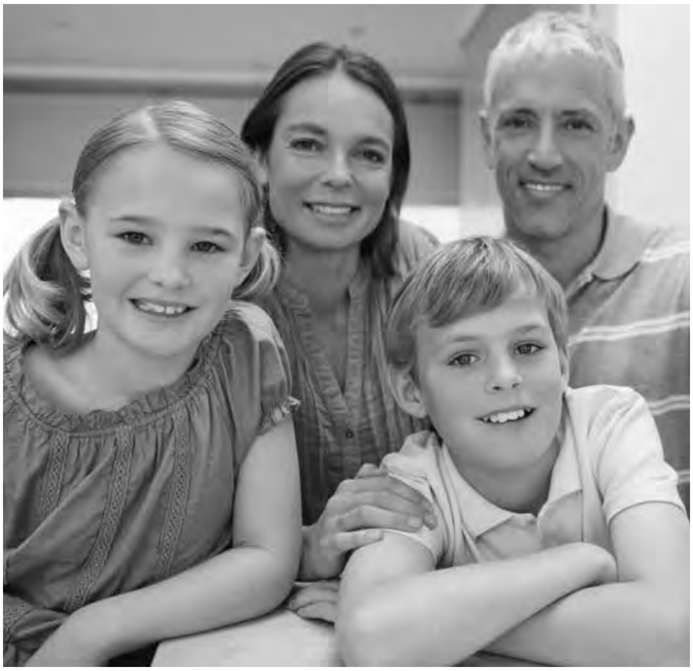

As you finish cancer treatment, you might be wondering: What happens next? The answer is different for every person. Some people return to the lives they were leading before their diagnosis, while the lives of others are significantly changed by their cancer experience. The challenge for every survivor is figuring out how to return to everyday life while adjusting to the effects of the disease and its treatment.

Recognizing these challenges and knowing how and when to ask for support can help you through this time of transition. Using this *ASCO Answers* guide may also be helpful. This booklet was designed to help survivors and their families and friends prepare for life after cancer treatment. Throughout this guide, you will find questions to ask your doctor, nurse, or another health care professional, as well as plenty of space to write down their answers or other important information. There are also workbook pages you can complete with the help of a member of your health care team to keep track of important information about your diagnosis, cancer treatment, and follow-up care.

However you choose to accurately keep track of this information, it is important for your future health to do so. Don't be afraid to ask questions or to let your health care team know you don't know what questions to ask.

My Health Care Team

Primary Care Doctor: ______________________________
Contact Information: ______________________________
____
____

Medical Oncologist: ______________________________
Contact Information: ______________________________
____
____

Radiation Oncologist: ______________________________
Contact Information: ______________________________
____
____

Surgeon: ______________________________
Contact Information: ______________________________
____
____

Oncology Nurse: ______________________________
Contact Information: ______________________________
____
____

Oncology Social Worker: ______________________________
Contact Information: ______________________________
____
____

OTHER SPECIALISTS:

Name: ______________________________
Specialty: ______________________________
Contact Information: ______________________________
____
____

Name: ______________________________
Specialty: ______________________________
Contact Information: ______________________________
____
____

Name: ______________________________
Specialty: ______________________________
Contact Information: ______________________________
____
____

Name: ______________________________
Specialty: ______________________________
Contact Information: ______________________________
____
____

Name: ______________________________
Specialty: ______________________________
Contact Information: ______________________________
____
____

What Is Survivorship?

Thanks to advances in medical research, the effectiveness of cancer treatment continues to improve. As a result, the number of people with a history of cancer in the United States has increased dramatically, from 3 million in 1971 to about 14 million today. As more people are surviving cancer, how long a person lives is no longer the only focus. It is also becoming increasingly important to determine how well survivors are able to live after treatment.

Survivorship means different things to different people, but it often describes the process of living with, through, and beyond cancer.

Defining survivorship

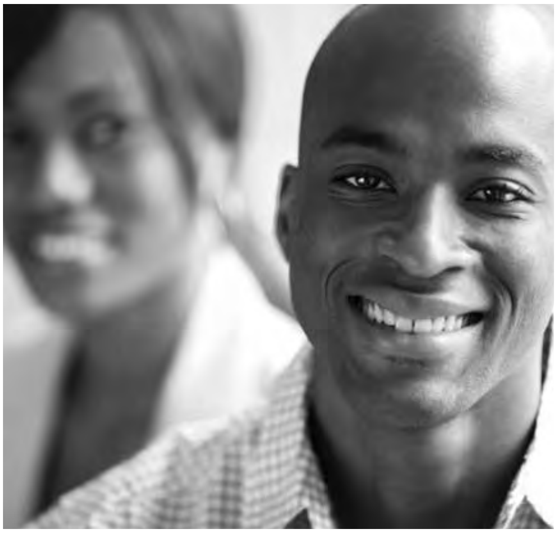

The word "survivorship" is often used in several different ways. One common definition is a person without cancer after finishing treatment. Another common definition is the process of living with, through, and beyond cancer. According to this definition, cancer survivorship begins at diagnosis and includes people who continue to receive treatment to either reduce the risk of the cancer coming back or to manage chronic disease.

No matter how it is defined, survivorship is unique for every person. Everyone has to find his or her own path to navigate the changes and challenges that arise as a result of living with cancer.

MY DEFINITION OF SURVIVORSHIP

____

____

____

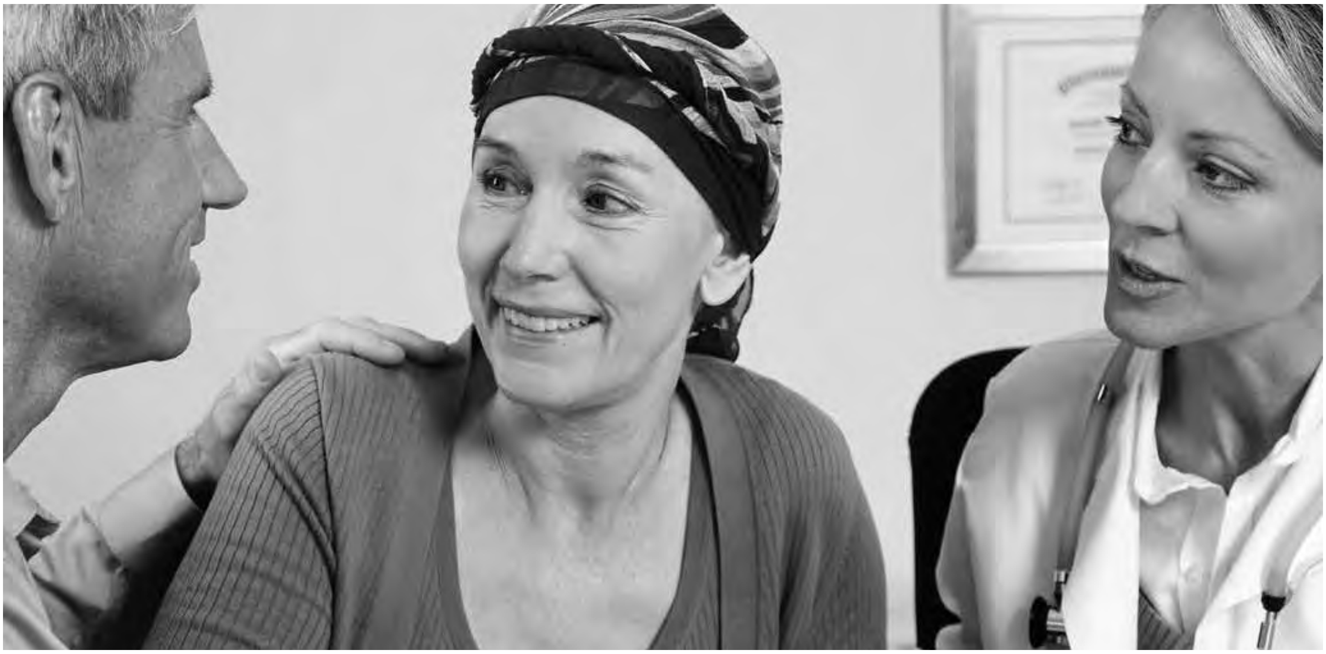

QUESTIONS TO ASK ABOUT SURVIVORSHIP

- Will I need to have any additional treatment after active treatment has finished?
- Who will be part of my health care team after active treatment ends, and what will each member do?
- Are there groups or online resources you would recommend to help me learn more about survivorship for my specific diagnosis?
- Whom should I contact if I have any questions or concerns?

NOTES:

Follow-Up Care

Cancer care does not always end when active treatment finishes. After cancer treatment has ended, your doctor may continue to monitor your recovery, manage any lingering side effects, and check to make sure the cancer has not returned. Your follow-up care plan may include regular physical examinations and/or medical tests during the coming months and years.

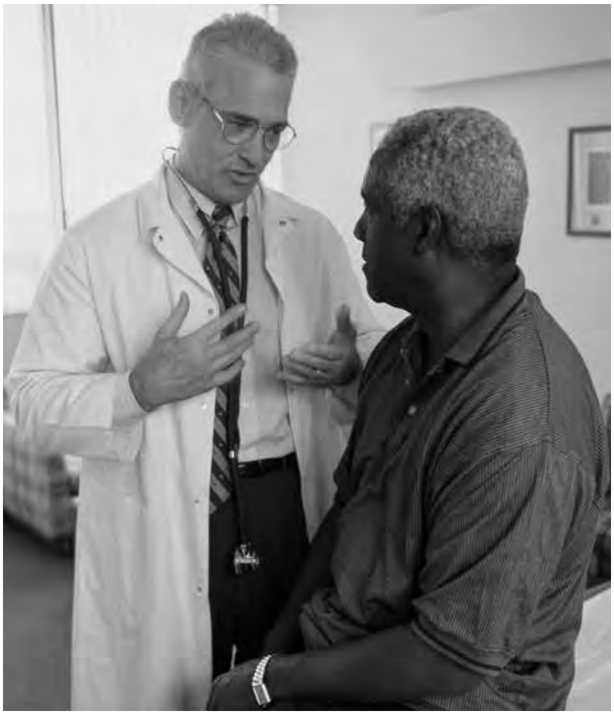

Talk with your doctor or clinician about any concerns you have about your future health. He or she can give you information and tools to help you immediately after cancer treatment has ended and for the long term. This is also a good time to determine who will lead your ongoing medical care. Some survivors continue to see their oncologist, while others see their family doctor or another health care professional. This decision depends on several factors, including the type and stage of the cancer, treatment side effects, health insurance rules, community resources, and your personal preferences.

Participating in follow-up care and keeping a medical support system in place are essential for maintaining both your physical and emotional health. It also helps many survivors feel in control as they transition back into their everyday lives. If you have any concerns about following the recommended follow-up care plan, talk with your doctor or another health care professional. You have been through a lot, and follow-up care can help you stay healthy into the future.

Watching for recurrence

One goal of follow-up care is to check for a recurrence of cancer. A recurrence is when the cancer comes back after treatment. Cancer may recur because small areas of cancer cells may remain undetected in the body. Over time, these cells may increase in number until they show up on test results or cause signs or symptoms. Depending on the type of cancer, this can happen weeks, months, or even many years after the original cancer was treated.

A recurrence may be local, which means the cancer has come back in the same part of the body where the original cancer was located; regional, which means it has returned in an area near the original location; or distant, which means it has returned in another part of the body. It is important to know that if a cancer recurs far from the location of the original cancer, it is still named for the part of the body where the original cancer began. For example, if a woman treated for breast cancer now has cancer in her liver, doctors will say she has metastatic breast cancer (breast cancer that has spread to another part of the body), not liver cancer.

The chance that a cancer will recur and the most likely timing and location of a recurrence depend on the type of cancer you were originally diagnosed with. Unfortunately, it is impossible for doctors to know who will experience a recurrence. Still, a doctor or clinician familiar with your medical history can give you more personalized information about your risk of recurrence and possibly suggest ways to minimize this risk.

To help find signs of a potential recurrence, your doctor or clinician will ask specific questions about your health and usually do a careful physical examination during your follow-up visits. Some people may also have blood tests or imaging tests. Your follow-up care will be based on the treatment and care recommendations for your specific diagnosis. In addition, your doctor may tell you to watch for specific signs or symptoms of recurrence.

If a recurrence is suspected, your doctor may order additional diagnostic tests, such as blood and/or urine tests, imaging tests, or biopsies, to learn as much as possible about the recurrence. After testing is done, you and your doctor will talk about the results and what the next steps should be.

SIGNS OR SYMPTOMS I SHOULD REPORT RIGHT AWAY

____

____

____

____

QUESTIONS TO ASK ABOUT CANCER RECURRENCE

- How likely is it that the cancer will return?
- What symptoms may be signs of a potential recurrence? Which should I report to you right away? Which should I report at my regular follow-up visits?
- What can I do to lower my risk of the cancer coming back after treatment?

NOTES:

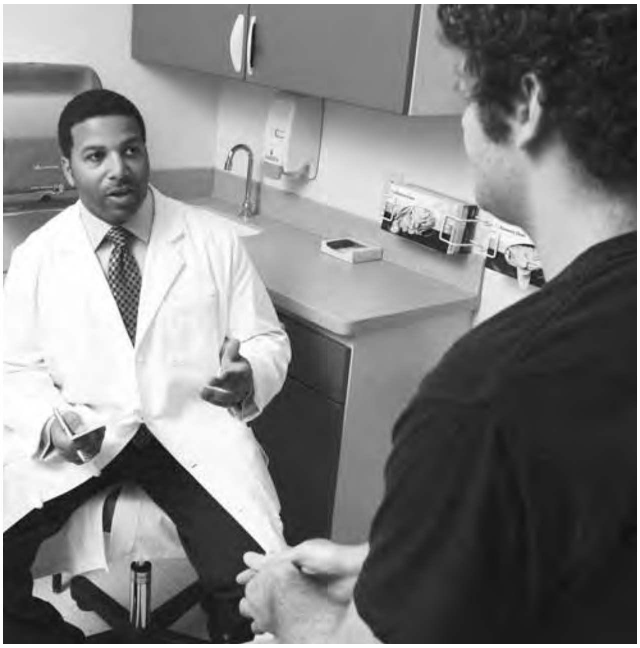

BILL WIPPERT

Managing late and long-term side effects

Most people expect to experience side effects during treatment. However, it is often surprising to survivors that some side effects may linger after treatment, called long-term side effects, or that other side effects may develop months or even years later, called late effects. Other health conditions you may have, such as diabetes or heart disease, may also be made worse by cancer treatment. These long-term effects are specific to certain types of treatment and usually develop within a defined time.

Your doctor will be able to tell you if you are at risk for developing any late effects based on the type of cancer you had, your individual treatment plan, and your overall health. However, some of the potential long-term side effects of cancer treatment are described here.

Bone, joint, and soft tissue problems. Cancer survivors who received chemotherapy, steroid medications, or hormonal therapy may develop thin or weak bones, called osteoporosis, or experience joint pain. You can lower your risk of osteoporosis by avoiding tobacco products, eating foods rich in calcium and vitamin D, and doing some types of regular physical activity. Your doctor may also prescribe medications that slow the rate of bone thinning, reduce new bone damage, and may promote bone healing.

Chemobrain. Cancer survivors commonly use the term "chemobrain" to describe difficulty thinking clearly after cancer treatment. However, survivors who do not receive chemotherapy may also report similar symptoms. The effects of chemobrain vary in severity and sometimes make it hard to complete daily activities. People who experience severe problems concentrating, multitasking, or understanding or remembering things should talk with a member of the health care team to learn about ways to manage these issues.

Digestion problems. Chemotherapy, radiation therapy, and surgery may affect how well a person digests food. For example, surgery or radiation therapy to the abdominal area can cause scarring, chronic pain, and intestinal problems that affect digestion. Additionally, some survivors may have chronic diarrhea that reduces the body's ability to absorb nutrients. If you experience poor digestion, a dietitian can provide personalized nutrition advice and eating plans. These might help you get enough nutrients and may help you return to a healthy weight.

Endocrine (hormone) system problems. Some types of cancer treatment may affect the endocrine system, which includes the glands and other organs that are responsible for making hormones and producing eggs or sperm. For example, men and women who receive radiation therapy to the head and neck area may have lower levels of hormones or changes to the thyroid gland.

Surgical removal of a woman's ovaries, chemotherapy, hormonal therapy, and radiation therapy to the pelvic area may cause a woman to have lighter and fewer regular menstrual periods or stop menstruating completely. Menstrual periods may return for some younger women after treatment, but women older than 40 are less likely to have their menstrual periods return. In some cases, although cancer treatment does not immediately cause

menopause, it may still cause menopause to start sooner than normal. Men with prostate cancer who receive hormonal therapy or who have their testicles removed may experience symptoms similar to menopause.

Emotional difficulties. Cancer survivors often experience a range of positive and negative emotions, including relief, a sense of gratitude to be alive, fear of recurrence, anger, guilt, depression, anxiety, and isolation. Survivors, caregivers, family, and friends may also experience post-traumatic stress disorder, which is an anxiety disorder that may develop after experiencing an extremely frightening or life-threatening situation. Talk with a nurse, social worker, or another member of your health care team if you are finding it difficult to cope with your emotions or if they begin to negatively affect your daily activities or relationships.

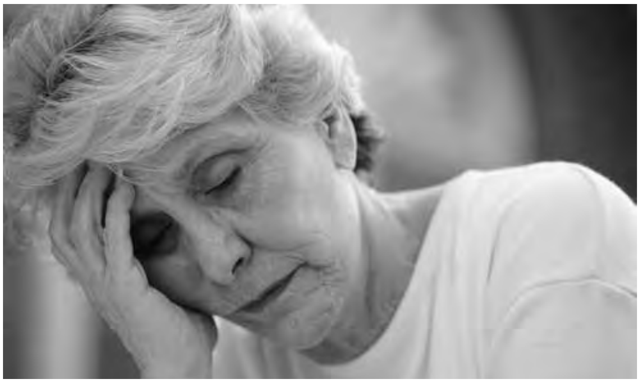

Fatigue. Fatigue is a persistent feeling of physical, emotional, or mental tiredness or exhaustion. It is the most common side effect of cancer treatment, and some cancer survivors experience fatigue for months and sometimes years after finishing treatment. Fatigue can seriously affect all aspects of a person's life, from relationships with friends and family to the ability to perform at work. It is important to tell your doctor if you are experiencing fatigue because there may be things your health care team can do to help.

Heart problems. Heart issues are most often caused by radiation therapy to the chest and specific types of chemotherapy. People 65 or older and those who received higher doses of chemotherapy have a higher risk of developing heart problems, which may include swelling of the heart muscle, problems with the heart's ability to pump blood, or heart disease. Talk with your doctor or clinician to find out if you should have regular evaluations for heart issues.

Lung problems.
Chemotherapy and radiation therapy to the chest may also damage the lungs, causing changes in how well the lungs work, thickening of the lining of the lungs, inflammation, and difficulty breathing. Cancer survivors who received both chemotherapy and radiation therapy may have a higher risk of lung damage. People with a history of lung disease and older adults may experience additional lung problems.

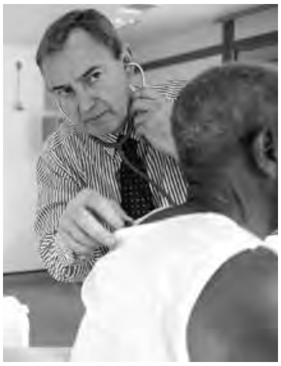

Lymphedema. Lymphedema is the abnormal buildup of fluid in soft tissue caused by a blockage in the lymphatic system. Most often, lymphedema affects the arms and legs, particularly in people treated for breast cancer or cancers that affect the urinary tract, bladder, kidneys, prostate, testicles, and penis, called genitourinary cancers. However, lymphedema can also occur in other parts of the body, including the breast, below the chin, in the face, and, less often, inside the mouth. In some cases, the swelling goes away on its own as the body heals and normal lymph fluid flow resumes. Lymphedema may become chronic when the lymphatic system can no longer meet the body's demands for fluid drainage. There is no cure for chronic lymphedema; however, there are ways to manage it.

Peripheral neuropathy. Peripheral neuropathy is a type of nerve damage that develops when the nerves that carry information back and forth between the brain and spinal cord are damaged. This damage can be caused by radiation therapy, some types of chemotherapy, or by the cancer itself. Depending on which nerves are affected, a person can develop numbness, tingling, pain, muscle weakness, constipation, or dizziness. If you develop neuropathy, your

health care team may be able to help treat your symptoms. Many people recover over the course of many months or a few years. Sometimes, the condition may be more difficult to cure and may require long-term management.

Secondary cancers. A different type of cancer may develop as a late effect of treatment with chemotherapy and radiation therapy, although this is relatively uncommon. Chemotherapy and radiation therapy can also damage bone marrow stem cells and increase the chance of developing either myelodysplasia, which is a blood cancer where the normal parts of the blood are either not made or are abnormal, or acute leukemia.

Other physical effects. Some survivors may have had a part of their body altered or removed as part of treatment. Survivors of cancers of the bone and soft tissue may experience physical and psychological effects from losing all or part of a limb, such as phantom limb pain, which is a feeling of pain that appears to be in the limb that was removed. To help survivors cope with these side effects and maximize their physical abilities, there are palliative care and rehabilitation services available (see page 30).

If you had a treatment known to cause specific late effects, your doctor may recommend scheduling regular physical examinations, scans, or blood tests to help identify and manage them. Talk with your doctor or clinician about which tests are recommended based on your treatment plan and how often you should have them. You can write down this information on page 13.

For more information about the possible long term effects of cancer treatment, visit **www.cancer.net/lateeffects.**

QUESTIONS TO ASK ABOUT LATE AND LONG-TERM SIDE EFFECTS

- What can be done to manage any side effects that continue after treatment?
- What are the most common late and long-term effects that may develop based on my treatment plan?
- What should I do if I notice a late effect?
- What screening tests do you recommend based on my cancer history?
- Are there other doctors or specialists I should see, such as a cardiologist or endocrinologist?

NOTES:

Keeping a personal health record

As time passes, it can be difficult to remember every detail of your diagnosis and treatment plan. At the same time, this information is very valuable to the doctors and clinicians who will care for you throughout your lifetime. To keep track of the most important information about their diagnosis and treatment, many survivors fill out a cancer treatment summary with the help of a member of their health care team and keep it with their health records.

Many survivors also ask their doctors and clinicians for a survivorship care plan or follow-up care plan. This serves as a guide for monitoring and taking care of survivors' health in the future. This plan is usually based on medical guidelines for a specific diagnosis, as well as a person's individual needs and preferences.

On pages 12 and 13 you will find everything you need to start creating a cancer treatment summary and survivorship care plan with the help of the staff at your doctor's office. Keep this information in your personal health record, and share it with your current and future health care providers. Now is also a good time to review your other medical documentation, such as your advance directive, to make sure that it reflects your current and long-term wishes. To learn more about putting your health care wishes into writing, visit www.cancer.net/advancedirectives.

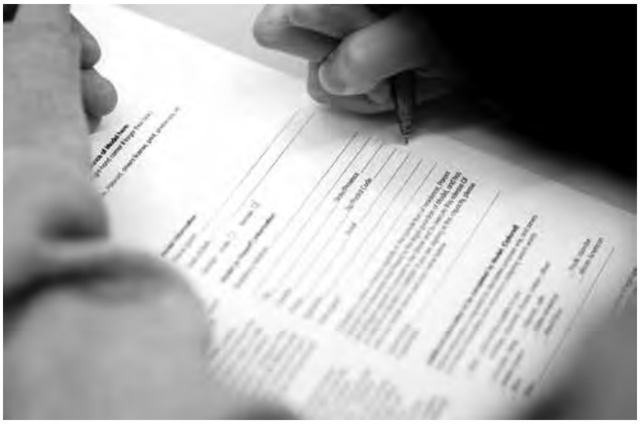

For cancer specific treatment summary forms and follow-up care plans, visit **www.cancer.net/treatmentsummary.**

QUESTIONS TO ASK ABOUT YOUR FOLLOW-UP CARE PLAN

- Who will be leading my medical care after I finish treatment?
- How do you usually provide survivorship care?
- Are there any survivorship clinics, survivorship centers of excellence, or other survivorship resources you would recommend?
- How often should I return for a follow-up visit with you? With my family doctor?
- What tests will I need during my follow-up visits?
- Can you provide me with a summary of my cancer treatment?

NOTES:

Treatment summary and survivorship care plan

Use these pages to keep track of the major aspects of your cancer treatment and the recommendations for your follow-up care. Please remember, this is not a comprehensive record of your care. Talk with a member of your health care team if you have any questions.

BACKGROUND INFORMATION

Family history of cancer: ☐ Yes ☐ No

Genetic/hereditary risk factor(s) or predisposing conditions:

____

____

____

____

Genetic counseling: ☐ Yes ☐ No

Genetic testing results: __________________________

____

____

____

DIAGNOSIS

Cancer type/subtype/location: ________________________________

____

Stage: ☐ I ☐ II ☐ III ☐ IV ☐ Not applicable

Diagnosis date (year): ___________

TREATMENT

SURGERY ☐ Yes ☐ No

Surgery date(s) (year): ___________

Surgical procedure/location/findings:

____

____

____

____

____

End date (year): ___________

Body area treated:

____

____

____

____

____

SYSTEMIC THERAPY ☐ Yes ☐ No

(chemotherapy, hormonal therapy, immunotherapy, other)

Name of drug	Start date	End date

RADIATION THERAPY ☐ Yes ☐ No

Symptoms or side effects that have continued after finishing treatment:

- ☐ Buildup of fluid in arms, legs, or neck (lymphedema)
- ☐ Change in mood or depression
- ☐ Change in weight
- ☐ Fatigue
- ☐ Heart issues
- ☐ Loss of appetite
- ☐ Low red blood cell count (anemia)
- ☐ Low white blood cell count (neutropenia)
- ☐ Memory or concentration loss
- ☐ Menopausal symptoms, such as irregular or stopped periods or hot flashes
- ☐ Sexual problems
- ☐ Skin changes
- ☐ Tingling, numbness, or pain in hands/feet (neuropathy)
- ☐ Other: ______________________

____

____

____

FOLLOW-UP CARE PLAN

Need for ongoing (adjuvant) treatment for cancer: ☐ Yes ☐ No

Additional treatment name	Purpose	For how long

SCHEDULE OF FOLLOW-UP VISITS

Doctor's name	When/how often

CANCER SURVEILLANCE OR OTHER RECOMMENDED TESTS

Test/procedure	When/how often

It is important to continue to see your primary care doctor for all general health care recommended for a person of your age, including screening tests for other cancers, when appropriate. You should also tell your doctor about:

1. Anything that could be a brand new symptom
2. Anything that continues to be a persistent symptom
3. Anything you are worried about that might be related to the cancer coming back

Signs or symptoms to tell the doctor about right away: ______________________________

____

____

Possible late and long-term effects: ______________________________

____

____

What concerns do you have as you transition into survivorship?

☐ Emotional and mental health
☐ Fatigue
☐ Fertility
☐ Financial advice or assistance
☐ Insurance
☐ Memory or concentration loss
☐ Parenting
☐ Physical functioning
☐ Sexual health
☐ School/work
☐ Stopping smoking
☐ Weight changes
☐ Other: ______________

____

____

____

Talk with a member of your health care team to find out how you can get help coping with these concerns.

Life After Treatment: What to Expect

In some ways, moving from active treatment to survivorship is one of the most complex aspects of the cancer experience because it is different for every person. After treatment ends, cancer survivors often describe feelings ranging from relief to fear. Some survivors say they appreciate life more and have gained a greater acceptance of themselves. At the same time, other survivors become anxious about their health and uncertain of how to cope with life after treatment, especially when frequent visits to the doctor stop.

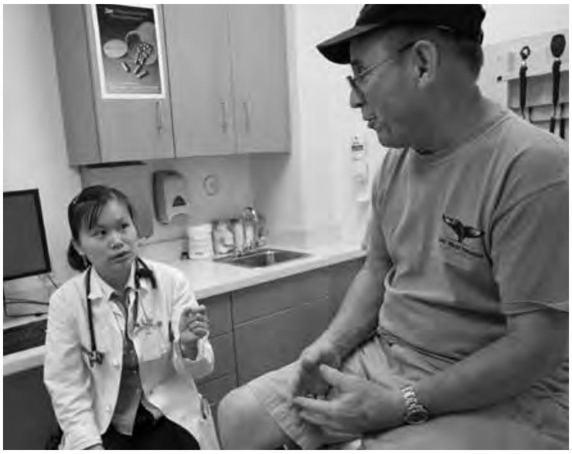
KAREN ELSHOUT PHOTOGRAPH

During treatment, people feel actively involved in their care, and the relationships they develop with their health care team can provide a sense of support and security. After finishing treatment, the safety net of regular, frequent contact with the health care team ends. Survivors often miss this source of support, especially because new anxieties and challenges may surface at this time, such as physical problems, emotional challenges, fertility concerns, financial issues, and workplace discrimination.

The transition to survivorship is often complex and is different for every person.

Fear of recurrence

One of the most common concerns survivors have is worrying the cancer will come back. The fear of recurrence is very real and entirely normal. Anxious thoughts like "Has the cancer come back?" and "What will happen if it does?" may be triggered by the appearance of a headache, cough, or joint stiffness, or it may come up before events like your diagnosis anniversary or follow-up appointments and tests. For some survivors, these worries and feelings of uncertainty lead to struggles with depression and anxiety. However, it is important to remember that although you cannot directly control whether the cancer returns, you can control how much the fear of recurrence affects your life.

WAYS TO COPE

Living with uncertainty is never easy, so it's important to remind yourself that fear and anxiety are a normal part of survivorship. Worrying about the cancer coming back is usually most intense the first year after treatment, but it generally gets better over time. Here are a few ideas to help you cope with the fear of recurrence.

Talk with your health care team. Although it's difficult to think about, every survivor needs to prepare themselves for the possibility that the cancer might come back. However, there is also no need to worry yourself unnecessarily. That is why getting accurate information about the risk of recurrence for your type and stage of cancer is extremely important. In general, most cancers have a predictable pattern of recurrence. No one can predict exactly what will happen in the future, but a health care professional familiar with your medical history can give you information about the chance the cancer might come back and what symptoms to look for. Keeping up with a regular schedule of follow-up visits can also provide a sense of control.

Recognize your emotions. Many people try to hide or ignore "negative" feelings like fear and anxiety. However, ignoring them may allow them to intensify and become overwhelming. It often helps to talk about your fears and feelings with a trusted friend, family member, or mental health professional. Or you can try writing down your thoughts in a journal, on a blog, or on social media. Talking and thinking about your concerns can help you explore the issues that underlie your fear. This might include the fear of having to repeat cancer treatment, losing control over your life, or facing death.

Take care of yourself. Healthy habits like eating nutritious meals, exercising regularly, and getting enough sleep help people feel better both physically and emotionally. You may also feel like you have more control over your health if you choose to avoid unhealthy habits, like smoking and drinking alcohol excessively, that have been linked to an increased risk of cancer recurrence.

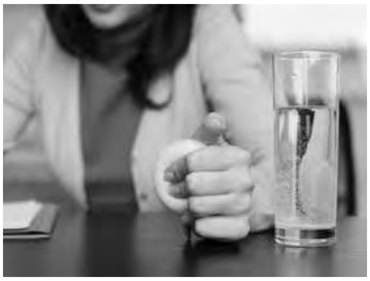

Reduce stress. Finding ways to manage your stress will help lower your overall level of anxiety. Try different ways of reducing stress to find out what works best for you. This could include spending time with family and friends, rediscovering old hobbies, doing activities you enjoy, taking a walk, meditating, enjoying a bath, exercising, or laughing at a funny book or movie. For more suggestions, see page 17.

Despite your best efforts to cope, you might find yourself overwhelmed by fear or anxiety. If this occurs, talk with your doctor, clinician, or nurse and consider a referral for counseling.

QUESTIONS TO ASK ABOUT COPING WITH FEAR AND ANXIETY

- What can I do to support my emotional health during my transition into survivorship?
- What are some healthy ways for me to deal with my feelings?
- What support services are available to me? To my family?
- What are some things I can do to manage my fears?

NOTES:

A new perspective on your health

For many people, the transition to survivorship serves as strong motivation to make positive lifestyle changes. Although cultivating healthy habits is a good idea for anyone, it is especially important for cancer survivors. This is because, as mentioned previously, survivors are often at higher risk for developing other health problems as a result of their cancer treatment. Healthy behaviors can help survivors regain or build strength, reduce the severity of side effects, reduce the risk of developing secondary cancers or other health issues, and enjoy life more.

If you decide to make changes to your lifestyle, it is important to set realistic goals and to recognize that change does not happen overnight. By setting your mind on accomplishing small, achievable goals each day, you will be working toward the larger lifestyle changes you want to achieve. Talk with your health care team about specific lifestyle changes you might want to pursue. These could include:

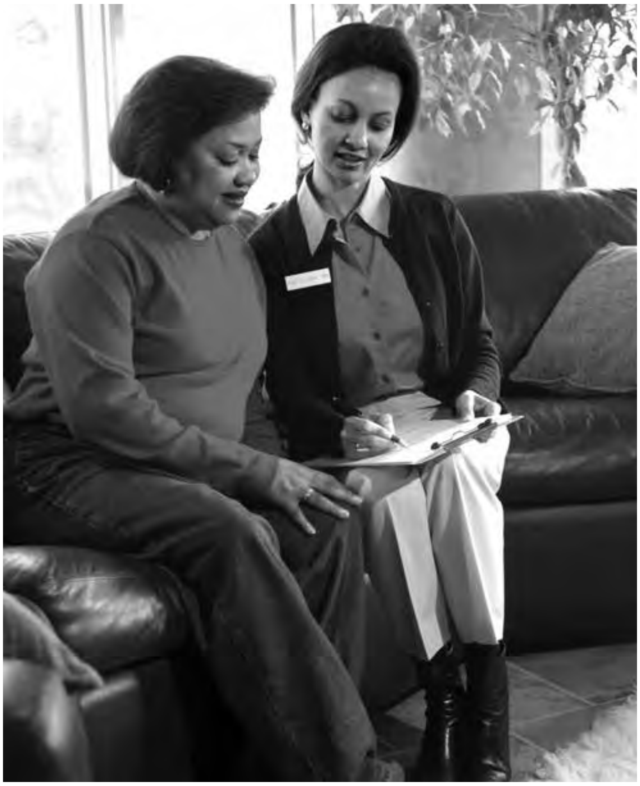

☐ Stopping tobacco use

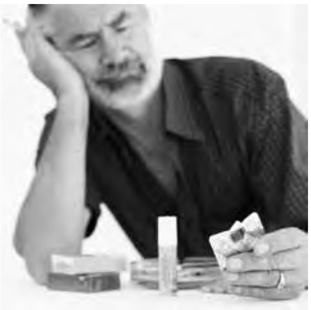

Stopping tobacco use is the single most important change a person can make to lower future cancer risk. Tobacco is linked to an increased risk of at least 15 types of cancer. If you smoke or use tobacco of any kind, making an effort to quit can also improve your recovery and overall health. Exposure to secondhand smoke is also dangerous, so other members of the household should be encouraged to quit smoking, too. Many resources are available to help, including medication and counseling, and can be found at www.cancer.net/tobacco.

Recommendations or referral: ______________________

____

____

____

____

☐ Reducing alcohol intake

In addition to tobacco, alcohol is another substance consistently linked to cancer. In general, experts recommend that women have no more than one alcoholic drink per day and men consume no more than two. One drink is defined as 12 ounces (oz) of beer, 5 oz of wine, or 1.5 oz of 80-proof liquor.

Recommendations or referral: ______________________

____

____

____

____

☐ Eating healthier

Choosing to eat meals filled with fresh fruits and vegetables, whole grains, and other unprocessed, low-fat foods may help cancer survivors regain strength after treatment. Nutritious eating can also reduce the risk of heart

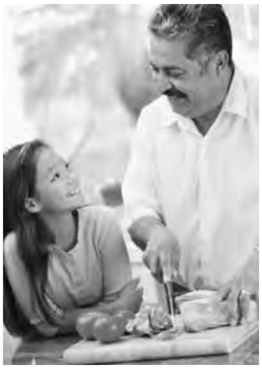

disease, high blood pressure, obesity, and diabetes. In addition, recent research suggests that some cancer survivors who make healthy food choices may have a lower risk of recurrence and live longer. Although most of these studies have focused on breast cancer, researchers have also noted these benefits in colon cancer and prostate cancer survivors who eat healthy diets. A dietitian can help you understand your nutritional needs, make healthy eating choices, and create tasty and appropriate meal plans.

Recommendations or referral: ______________________
____
____
____
____

☐ **Exercising regularly**
The American College of Sports Medicine recommends that survivors avoid inactivity. Start slow and build up. Aim to do at least 150 minutes of moderate aerobic exercise, like walking, every week and resistance (strength) training two or three days per week.

Research is starting to link exercise with improved quality of life for cancer survivors. Regular physical activity can help survivors reduce anxiety, depression, and fatigue; improve self-esteem; increase feelings of optimism; improve heart health; reach and maintain a healthy weight; and boost muscle strength and endurance. Exercise also reduces the risk of high blood pressure, heart disease, stroke, and diabetes. In addition, some studies have shown that exercising regularly may help prevent the recurrence of breast, colon, prostate, and ovarian cancers.

Remember, even a small amount of physical activity is helpful. Talk with your doctor or clinician before you start an exercise program because you may need to adapt the types of exercises you do to your specific needs and limitations. A certified health and fitness professional can help you develop a plan based on your doctor's or clinician's recommendations, but this is not typically covered by insurance.

Recommendations or referral: ______________________
____
____
____
____

☐ **Managing stress**

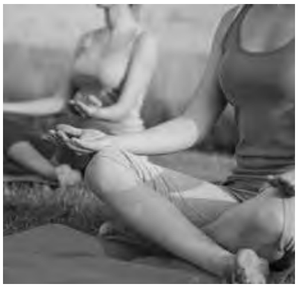

Being diagnosed with a serious illness like cancer is very stressful, and everyday life often adds to this stress. Learning how to manage stress is extremely important for your recovery. Experiencing high levels of stress for a long time has been linked to health problems and a lower quality of life. A big step in reducing stress can be made through small changes in your life, such as learning to say "no" to tasks you don't have the time or energy to complete, doing your most important tasks first, and getting help with potentially challenging issues such as finances.

Other ways to manage stress include exercise, social activities, support groups, mindfulness, acupuncture, yoga, tai chi, massage, and other relaxation techniques. Many relaxation techniques can be learned in a few sessions with a counselor or in a class.

Recommendations or referral: ______________________
____
____
____
____

☐ **Other:** ________________________________
____
____

Personal reflection

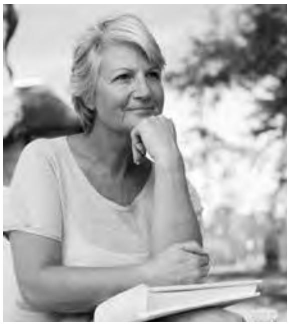

Many survivors struggle with questions of why they had cancer and why they survived when others have not. Some survivors may find themselves re-examining long-held views or beliefs as they try to make sense of their experience and find new meaning in life. Although every person creates meaning from their experiences in their own way, other cancer survivors have said they have found answers to some of these difficult questions by:

Re-evaluating old patterns and priorities. Some survivors look at their cancer experience as a "wakeup call" and begin to ask questions like: Are my current roles in my family or as a friend fulfilling? Does my job make me happy or am I just doing what other people expect me to do? What are the most important things in my life now?

Reaching out for spiritual support. For some, spirituality and faith are a source of comfort and guidance. Many hospitals and cancer centers have chaplains who can give support to people of all faiths, as well as those who don't consider themselves religious at all. Local cancer organizations may also be able to help you find religious or spiritual leaders in your community who have experience helping cancer survivors.

Keeping a journal or blogging. Writing down your thoughts and feelings starts a process of self-discovery and, for some, of spiritual development. Allowing yourself to think every day or every week about your feelings is a way to get to know yourself better and to understand what gives meaning to your life now. Because blogging is much more public than journaling, it may also connect you with, and help inspire, other people who are going through a similar situation.

Finding new ways to support emotional well-being. Some survivors choose to begin new activities, such as yoga, meditation, drawing, and music therapy, that help support their spiritual and emotional health and make them feel less fearful and anxious. Other people become more active on social media as a way to gather information and cope.

QUESTIONS TO ASK ABOUT REFLECTION

- How can I manage feelings like guilt, confusion, or anger?
- Are there religious or spiritual organizations in my community that provide specialized support or services for cancer survivors?
- Where else can I find emotional or spiritual support?

NOTES:

Changes within families and relationships

Cancer often changes the way you relate to your family, partner, and friends, and the way they relate to you. When active treatment is over, some survivors need different types of support than they had before. Some friends may become closer, while others may distance themselves. Families can become overprotective or may have exhausted their ability to be supportive. Relationship problems that may have been ignored before a cancer diagnosis can be brought to the surface. Everyone is changed by the cancer experience in ways they may not even be aware of.

PARENTING AND FAMILY LIFE

Living with and beyond cancer often makes people rethink the way they live their lives, including the way they parent. When active treatment ends, many survivors find that parenting after cancer presents unique challenges.

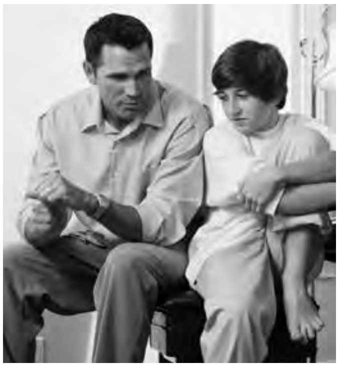

For months or even years, the demands of cancer and treatment may have made it difficult to spend time with your children. The guilt of constantly being away from home or unavailable can trigger a strong desire to just be a "normal parent," or sometimes even a "super parent," to make up for that lost time. However, long-term side effects of cancer treatment, such as memory loss, difficulty concentrating, pain, fatigue, or permanent disabilities, can make parenting even more demanding and frustrating. It is important not to compare yourself to other parents during this time. As you adjust to life after treatment, you can only do your best and shouldn't be hard on yourself because of what you might see as limitations.

Sometimes, making every minute after treatment with your family "count" becomes a top priority. However, trying to pack every minute of every day full of love and fun puts a lot of unnecessary stress and pressure on you, your partner, and your children. These attempts to overfill time may be exhausting for everyone and may do the opposite of what you are trying to accomplish. Instead, think about choosing more relaxed and manageable ways of connecting that will give you quality time and the chance to enjoy shared experiences, like watching a favorite movie together or going for a walk.

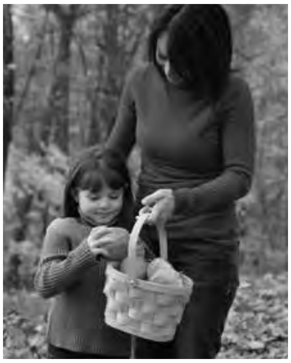

Fear of recurrence may be another strong factor in the way survivors parent. Worrying about having to go through treatment again and being separated from your children or not surviving cancer the next time can negatively affect your relationship with your children. Some parents pull away, while others may become overprotective. The best way forward is to focus on communicating openly and honestly with your family about your feelings and the chance the cancer might come back and encouraging them to share their feelings and concerns with you.

SEXUALITY AND INTIMACY

In general, although people are less interested in sex while having cancer treatment and at times of crisis, interest in sex usually improves during recovery and survivorship. However, some survivors may experience changes in their sexual function or sex drive caused by cancer and cancer treatment.

Some treatments, even those not directed at the pelvic area or groin, can cause physical side effects that interfere with sexual function. But even other physical changes not directly related to sexual function may affect the way a person feels about his or her body and his or her physical attractiveness, such as losing a testicle or a breast, needing a colostomy or another type of ostomy, losing weight or hair, or having scars or skin changes. Even a person

whose body was not outwardly changed by cancer may feel differently about his or her body. All of these changes affect self-image, self-confidence, and a person's sense of attractiveness.

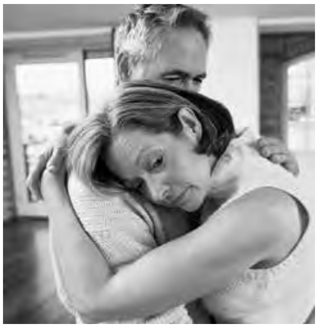

Even though it may feel awkward or uncomfortable, it is important to discuss what you are experiencing with a member of your health care team. If your doctor or clinician does not seem comfortable or experienced with these concerns, ask for a referral to a social worker or to someone else on the team who may be better equipped to provide help and support.

Intimacy is very closely connected to your feelings about your relationship as well as your feelings about yourself. Therefore, finding a counselor who has experience working with people with cancer and talking through some of these issues can help both you and your partner.

Good communication involves talking openly and honestly about your thoughts, feelings, and fears with someone who listens and supports you. Good communication includes not only sharing your own thoughts and feelings, but also listening to the other person's thoughts and feelings and accepting them without criticism or blame. By establishing open and ongoing communication with your spouse or partner, you both can better adapt to the changes cancer has caused in your lives as well as in your relationship.

QUESTIONS TO ASK ABOUT RELATIONSHIPS

- How can I be mindful of the ways cancer could affect the way I parent?
- If I have difficulty talking to my children, who can help?
- Could my treatment plan affect my sex life? If so, how and for how long?
- Can you refer me to another health care professional who can help facilitate discussions with my friends and family?

NOTES:

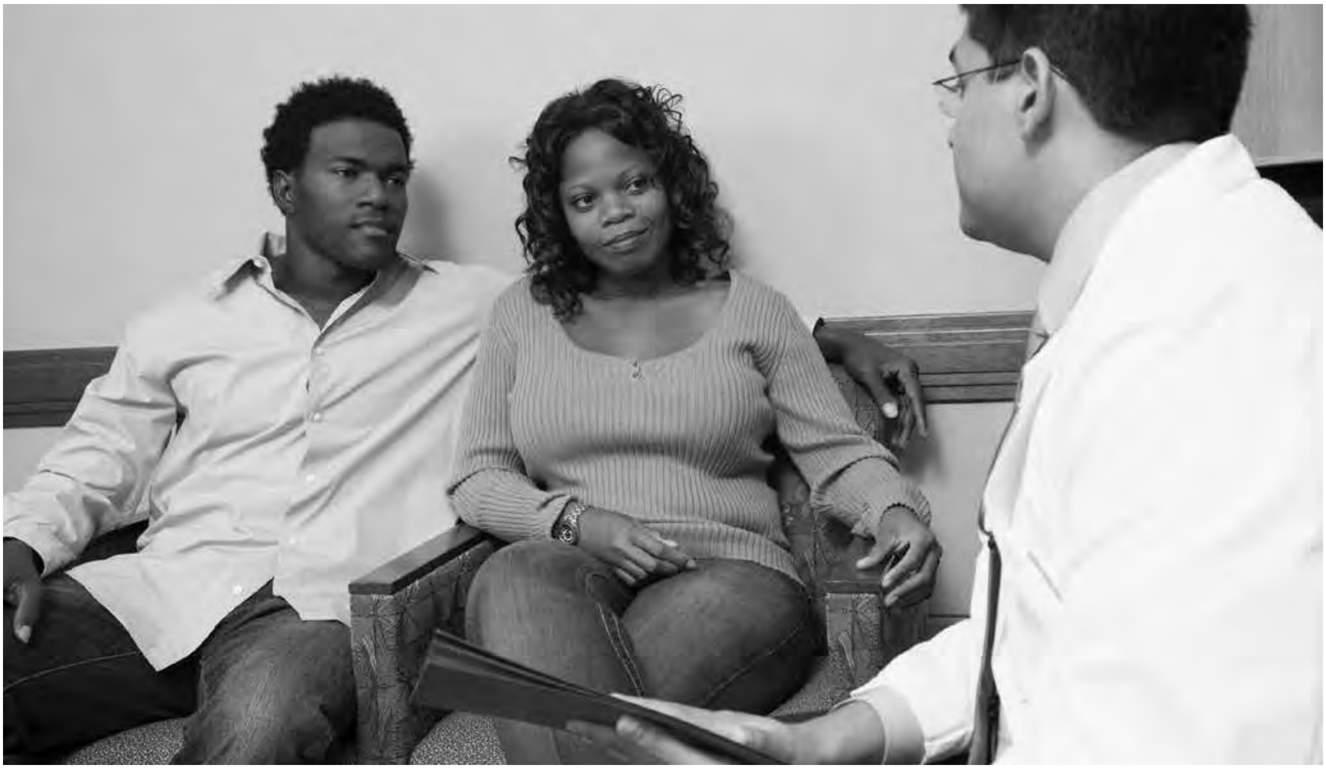

Starting or expanding your family

Deciding when and whether to have a baby is often a difficult decision for survivors. There are a number of emotional and physical factors that both survivors and their spouses/partners need to consider before starting or adding to their families.

In general, becoming pregnant after cancer treatment is considered safe for both the mother and the baby, and pregnancy does not appear to increase a woman's risk of recurrence. However, the exact amount of time female survivors should wait before trying to become pregnant depends on the type and stage of cancer, the type of treatment the woman received, any need for ongoing treatment, and her age and personal preferences.

If you are a female survivor, you also should talk with your doctor or clinician about whether your body can safely handle pregnancy. Sometimes, cancer treatments damage specific areas of the body, such as the heart or lungs. Before becoming pregnant, these organs may need to be checked to be sure pregnancy will be safe. You may be referred to an obstetrician who specializes in caring for women during and shortly after a pregnancy when they have other health concerns.

For male survivors, there are no specific guidelines for trying to have a child after finishing cancer treatment. However, doctors may recommend waiting before trying to have a child, with the exact amount of time depending on a number of factors.

DEALING WITH INFERTILITY

Some couples find that cancer treatment has made it difficult or impossible for them to have children, leading to intense grief and anger. It is important to find support as you cope with these changes and losses. Your doctor, clinician, social worker, or counselor may also be able to help you look into other ways to start or expand your family.

Assisted reproduction. Cancer treatment and age may reduce the number of eggs in a woman's ovaries, called the ovarian reserve, making it difficult for her to become

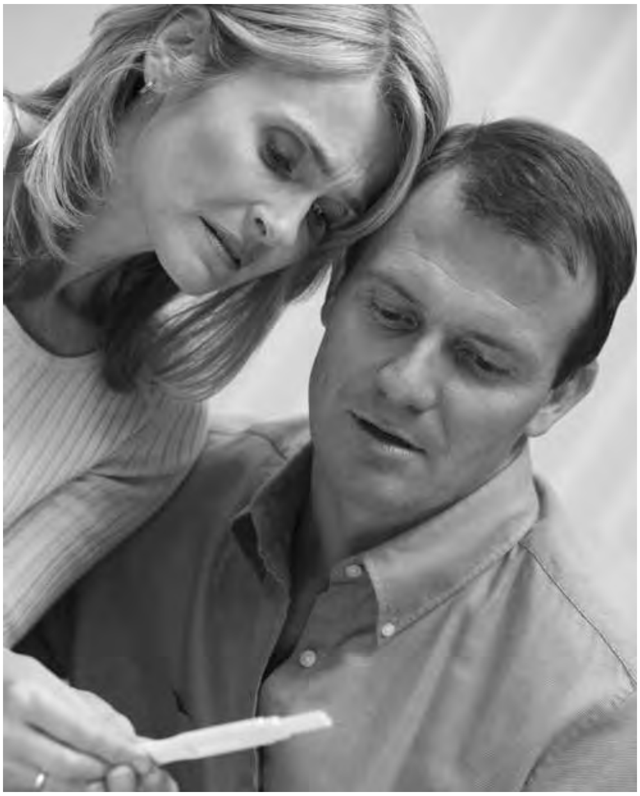

pregnant. If blood tests and ultrasounds show this has occurred, a fertility specialist may be able to use assisted reproduction techniques, such as in vitro fertilization (IVF). During this process, a woman's eggs or donor eggs (see below) are fertilized with sperm outside the body. One or more of the fertilized eggs (embryos) are later transferred into the woman's body to develop into a baby. For the pregnancy to be successful, the woman's uterus must be healthy, and she will have to take hormones before and after the procedure.

Donor eggs. Women with a low ovarian reserve may be able to use another woman's eggs to become pregnant. The donated eggs are fertilized in a laboratory with the sperm of the woman's partner or of a donor using IVF techniques.

By law, all egg donors, whether they are a family member, friend, anonymous donor, or known donor from an agency, are screened for psychological issues, medical conditions, and potential genetic diseases before they are allowed to donate eggs.

Donor sperm. If a man who had cancer treatment did not store his sperm before starting treatment, he can use sperm donated to a sperm bank by another man to become a father. In most cases, the sperm, which is screened for infectious diseases, is donated anonymously. However, sperm banks usually record the physical traits of the donor.

Testicular sperm extraction and epididymal sperm extraction. For men who do not have mature sperm in their semen, this procedure involves removing a small amount of tissue from the testicle. This tissue is examined under a microscope to find mature sperm, which can be frozen or used immediately for IVF.

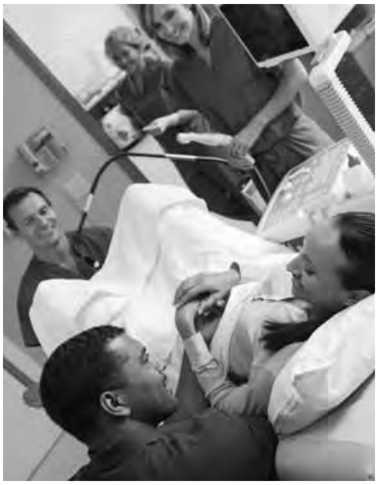

Donor embryos. Donor embryos usually come from couples who had an infertility treatment that resulted in extra embryos. As with egg donation, the embryos are then transferred into the uterus of the woman who wants a child, and the woman will have to take hormones before and after the embryos are inserted. Although a couple or individual who uses a donated embryo will not be genetically related to the child, the procedure allows a woman with a healthy uterus to experience pregnancy. By law, the couple donating the embryos must have the same tests that are required for the egg donation process.

Surrogacy and gestational carriers. If a woman is not able to carry a child, or if becoming pregnant could put her health at risk, having another woman carry the baby during pregnancy is an option. This is called surrogacy if the woman who will carry the child becomes pregnant through artificial insemination with the father's sperm. If an embryo created from the mother's egg and the father's sperm

is implanted into the woman, she is called a gestational carrier. Surrogacy and gestational carrier laws are different in each state, so it is important to consult an attorney if you are considering this option.

Adoption. Adoption is the permanent, legal transfer of parental rights of a child from the biological parent to another couple or individual. Although most adoption agencies allow cancer survivors to adopt, some require a letter from a doctor certifying good health, and others may require a certain amount of time to pass after you have completed treatment for cancer. If the adoption is taking place outside the United States, there may also be international rules related to the health of the adoptive parents.

For more information about having a child after cancer, visit **www.cancer.net/survivorship/life after cancer.**

QUESTIONS TO ASK ABOUT HAVING CHILDREN

- Could cancer treatment affect my ability to become pregnant or have children?
- How long should I wait before trying to become pregnant or father a child?
- How will trying to have a child affect my follow-up care plan?
- Where can I find support for coping with fertility issues?
- Whom should I contact if I need help talking with my spouse or partner about fertility issues?

NOTES:

Going back to work

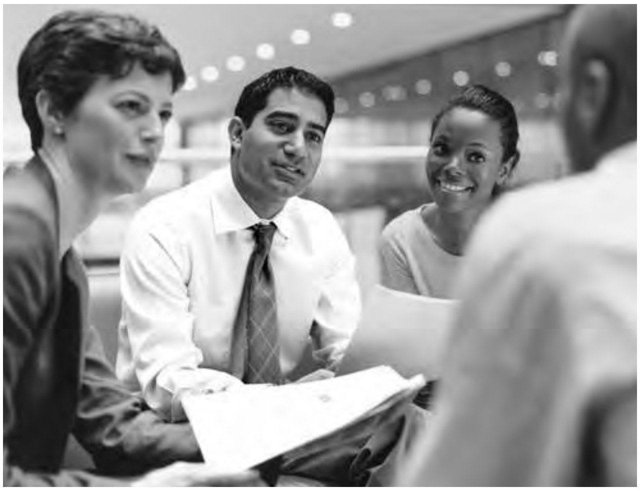

For many people, returning to a full-time work schedule is a sign both to themselves and the world that they are getting back to "normal." Working can provide opportunities to reconnect with colleagues and friends, focus on something other than cancer, get involved in interesting and challenging projects, and start settling back into a regular routine and lifestyle. However, transitioning back into the workforce may feel overwhelming at times.

Every survivor's work situation is different. Many people with cancer who took time off for treatment return to work afterwards, while many others may have worked throughout treatment. Still others may not be able to return to work because of long-term side effects. Your decisions about work will likely depend on your financial resources, health insurance, the type of work you do, and the nature of your recovery.

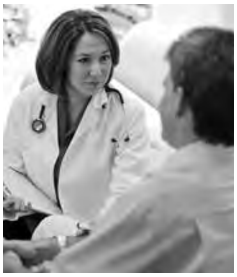

PLANNING YOUR RETURN

For survivors who cut back on their hours or stopped working, the first step is to talk with your doctor or clinician about whether you are ready to return to work. The timing depends on the type of cancer and treatment you had and the type of job you perform. If your job is stressful or physically demanding, you may need to wait longer before returning to work. Ongoing treatment or side effects, such as fatigue, may also cause delays.

Once you know it's okay to return to work, a good next step is to set up a telephone or in-person meeting with your human resources department to discuss transition plans. Ask whether your employer has a formal "return-to-work" or disability management program. You may also want to discuss the possibility of flexible work arrangements, such as part-time hours, partial or full-time telecommuting, job sharing, reassignment to another position, leave time for doctor visits, or periodic work breaks to take medications and contact members of your health care team. These are all considered reasonable accommodations under the Americans with Disabilities Act (ADA).

Before granting a reasonable accommodation, your employer may request documentation that verifies your limitations, such as fatigue, chronic pain, and cognitive difficulties, which are classified as disabilities under the ADA. However, employers are not allowed to ask for your medical records. An employer may deny a request if the accommodation would cause an "undue hardship," such as being too difficult or expensive to implement. However, an employer is required to determine if there is an easier or less costly accommodation that can be made to meet your needs.

Other things to consider to make your transition back to work smoother include:

- Planning to take small breaks throughout the day to help maintain your energy level
- Using lists and reminders or setting meeting and task alarms on your office e-mail system
- Scheduling frequent meetings with your manager to talk about the transition and make any necessary changes or adjustments

TALKING WITH COWORKERS

It's your decision when and how to tell coworkers about your cancer experience, if you even decide to talk about it at all. However, if you have been absent for a while or your

physical appearance has changed, some of your coworkers may have questions. It is important to decide what you want to tell people in advance and how you plan to do it. You may want to have private conversations with a few close coworkers. Or you may find it easier to send an e-mail or make an announcement at a staff meeting. Consider your work culture, what feels right to you, and your need for privacy versus your need for accommodations and support. You can also ask your manager, a close coworker, or a human resources professional to help you decide if, when, and how to tell others about your cancer.

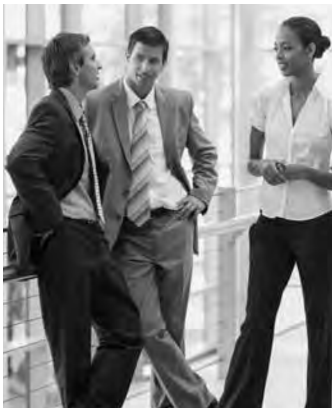

It always helps to keep your explanation simple and let people know how your return to work will affect them. For example, you might consider saying: "I've completed treatment for cancer, and I'm currently doing well. It's good to be back at work. Just so you know, I will be here 20 hours per week for the next 4 weeks, and I will return full time after that. In the meantime, Joe Smith will be covering projects A and B."

Some employers and coworkers may not respond well. Their reactions usually have to do with past experiences or a lack of familiarity with cancer. However, most survivors find that their coworkers are supportive and caring. People often take your lead; if you are comfortable talking about your experience with cancer, they will likely feel the same.

DEALING WITH DISCRIMINATION

Although many survivors can be as productive as they were before treatment, some find they are treated differently or unfairly. For example, some employers and colleagues may assume that a person's productivity will decrease or that performance will fall below the company's expectations. Other types of discrimination may include receiving a demotion for no clear reason, having an earned job promotion withheld, being overlooked for consideration in a new position, and finding a lack of flexibility in response to requests for time off for medical appointments.

It is important to understand that there are laws and regulations that prohibit discrimination, such as the ADA, and you can take legal action if necessary. However, it is often better to take steps to try to prevent discrimination when you return to work after cancer treatment by:

- Catching up on new projects or developments that occurred while you were gone
- Refreshing job skills, if necessary, by reviewing past work assignments or attending classes or workshops
- Seeking counseling from a professional about making the transition back to work
- Getting advice and tips from other cancer survivors through a support group
- Asking your doctor to write a letter stating your ability to return to work

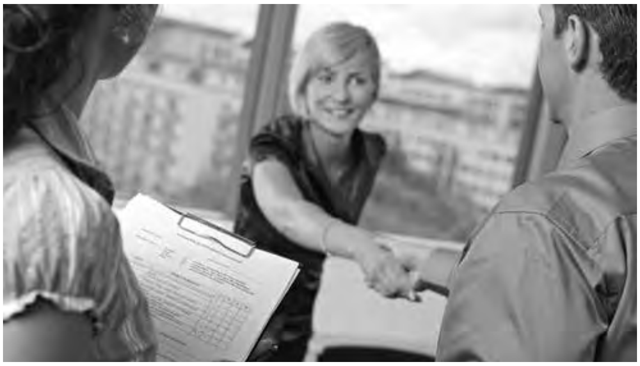

FINDING A NEW JOB

For some survivors, the cancer experience reshapes their career priorities and causes them to question whether they still want to continue on their current career path. Other survivors are unable to return to their previous jobs and must take their careers in a new direction. No matter what the reason, the thought of applying and interviewing for a new job may make you uneasy.

When applying and interviewing for a new job, some cancer survivors worry about how they will explain gaps in their work history or their reasons for leaving positions they

had before their cancer diagnosis or treatment. If you are concerned about explaining gaps in employment, it may be helpful to talk with a career counselor or social worker. He or she can teach you interviewing skills and help you organize your résumé by experience and skills instead of by date.

During the interview process, an employer, by law, cannot ask questions about your health or about a medical condition, such as whether you have had cancer. If you choose to tell a potential employer about your cancer history, the employer is still not allowed, by law, to ask you any questions about the cancer, the treatment you received, or your recovery. The employer also must keep any information you share about your history of cancer or any other medical information confidential. An employer is allowed to ask questions related to the specific duties and responsibilities of the job, such as whether you can lift a certain number of pounds or travel.

Some job seekers find encouragement in support groups, which help them connect with other people who are looking for work. These groups also provide suggestions and tips on networking with potential employers and can be good resources for learning about potential job openings. In addition, books and career counselors can provide guidance on how to network effectively. Taking proactive steps can boost your self-esteem and help you stay positive while you are looking for employment.

QUESTIONS TO ASK ABOUT EMPLOYMENT CONCERNS

- If I am nervous or feel overwhelmed about returning to work, who can help me?
- If I have difficulty talking with my coworkers about my cancer experience, what are some coping strategies I can try?
- What information do I need to give my employer when I return to work? What information can remain private?
- If I have on-the-job difficulties when I return after treatment, who can help me understand my legal rights?

NOTES:

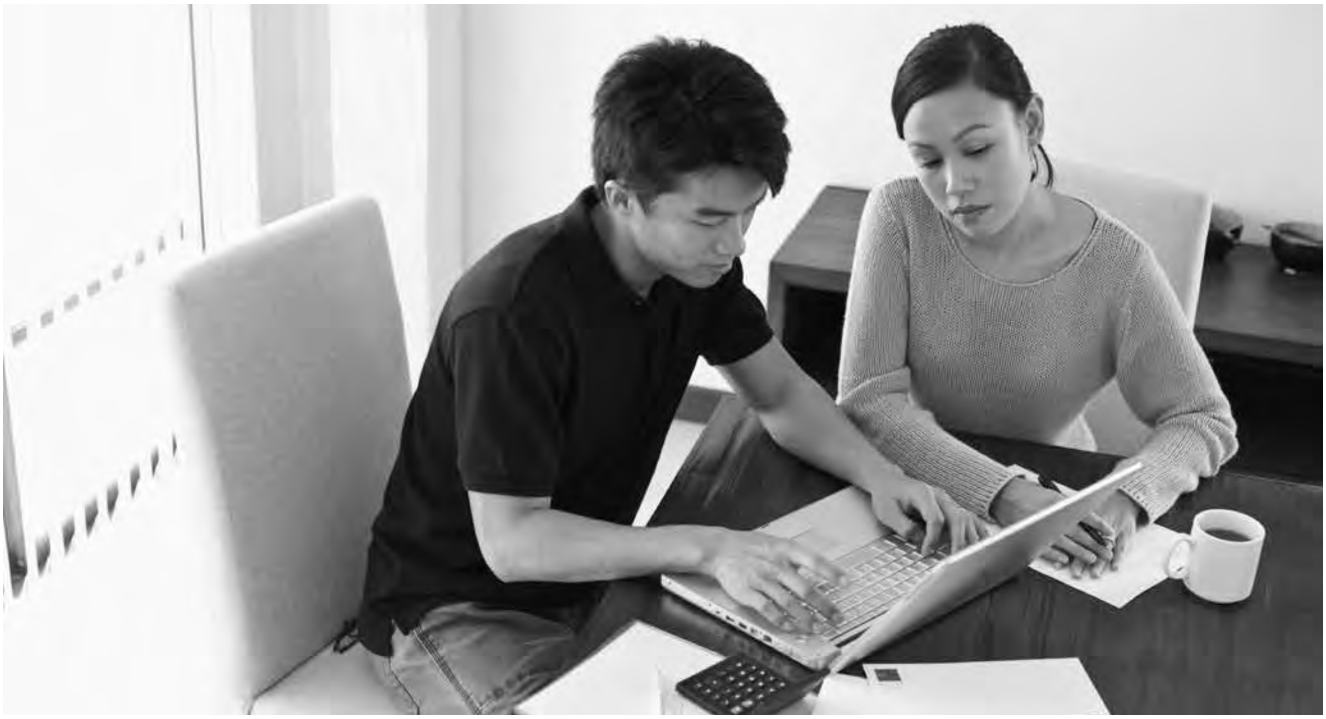

Managing your finances

The costs of cancer care can be high. Even people with reliable health insurance can be left with bills that quickly add up. Often, survivors have already lost income because they weren't able to work as much or at all during treatment, making it difficult to pay for both medical and household expenses. This financial stress may increase if you are unable to return to work after finishing treatment. Here are a few things to consider as you cope with the financial impact of cancer:

Investigate other sources of income if you are unable to return to work. If a disability or other long-term side effect of cancer treatment has made it impossible for you to return to work, there are a number of potential sources of income to consider, including long-term disability insurance, life insurance policies, and retirement plans. Some cancer survivors apply for Social Security Disability Insurance or Supplemental Security Income. To learn more about these federal programs, including eligibility requirements, call 800-772-1213 or visit www.ssa.gov/disability.

Organize bills and rank them in order of priority. As you open your bills, organize them into categories such as medical bills, household bills, credit card statements, taxes, etc. Then figure out how much money you have in your budget to put toward payments. Your rent or mortgage, utilities, taxes, and medical expenses should be at the top of your bill-paying list.

Make an appeal. If your insurance company has denied payment for a service or treatment, you have the right to ask them to conduct a full and fair review of its decision. If the company still denies payment after considering your appeal, the Affordable Care Act allows you to have an independent review organization decide whether to uphold or overturn the plan's decision.

Ask if the insurance payment can be considered "payment in full." If you are unable to pay for tests, procedures, and other treatments that were not completely covered by your insurance plan, making this request to the hospital, cancer center, or doctor's office is often more successful than people expect. Some hospitals have funds to offset medical services that aren't fully covered by insurance.

Talk to your creditors. Usually if you call and explain your situation, your creditors or a credit counseling service will work with you to create a payment plan. Most companies would prefer receiving small payments, even $10 a month, than nothing at all. Give the person you talk to a telephone number and the hours that you can be reached if there are any problems. Also ask them to communicate with you in writing.

Ask for help. If you can't face the stack of bills that has collected, ask a trusted friend or family member to help you organize them and plan your budget. You can also talk with an oncology social worker or patient navigator about your financial concerns. They can help connect you with financial assistance programs and other sources of financial aid.

Contact an organization that offers help for cancer survivors facing financial challenges. There are many national and local organizations that offer financial information, advice, and support for cancer survivors. Contact them directly to learn more about specific programs and services, including eligibility criteria.

For more advice and suggestions for managing the costs of cancer care, visit **www.cancer.net/managingcostofcare.**

QUESTIONS TO ASK ABOUT YOUR FINANCES

- How many more medical bills should I expect to receive related to my active treatment period?
- If my insurance claim is denied, who can help me file an appeal?
- If I am having difficulty paying my medical bills, are there organizations that can help?
- What resources and support are available to me if I am unable to return to work after finishing treatment?
- If I am having trouble paying for basic items, like food or heat, due to the cost of my cancer treatment, are there organizations that can help?
- Where can I get free or low-cost personal items after treatment, such as medical supplies, if needed?
- Who can help me understand what my insurance will cover in terms of my recommended survivorship care plan, such as follow-up exams and medical tests?

NOTES:

Support for Coping With Challenges

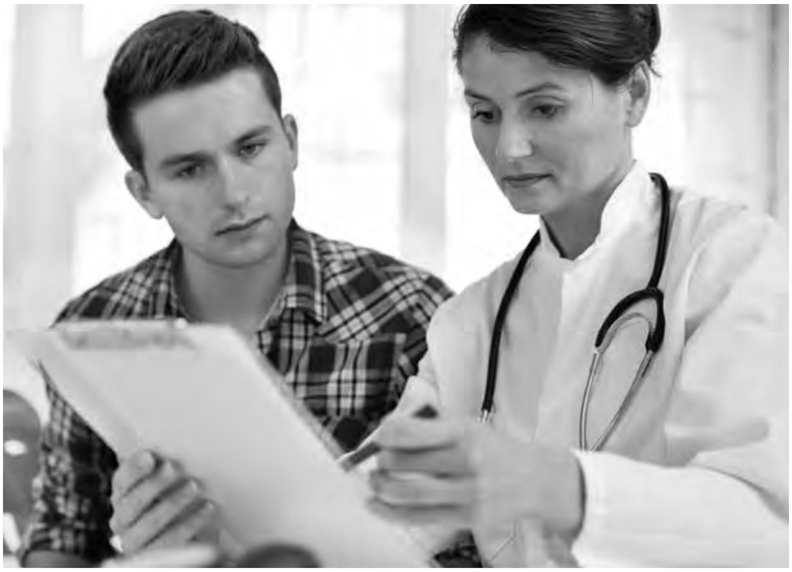

Every cancer survivor has individual concerns and challenges, some of which may not have been addressed specifically in this booklet. With any challenge, though, a good first step is being able to recognize your fears and talk about them. Effective coping requires understanding the challenge you are facing, thinking through solutions, asking for and allowing the support of others, and feeling comfortable with the course of action you choose.

Talking with your doctor, clinician, nurse, or another member of your health care team about any concerns you may have is an important part of your follow-up care, especially if something you are experiencing is holding you back from enjoying your life. Just as there were support options during treatment, there is also help for you during your transition into survivorship and beyond.

Cancer rehabilitation

Cancer rehabilitation helps people who have had cancer function at their best physically, socially, mentally, and professionally. The goal of rehabilitation is to help people regain control over many aspects of their lives and remain as independent and productive as possible. Rehabilitation is valuable for anyone living with cancer, those recovering from cancer treatment, and their families by:

- Improving physical strength to help offset any limitations caused by cancer and cancer treatment
- Helping the person become more independent and less reliant on caregivers
- Helping the person adjust to any losses caused by cancer and cancer treatment
- Reducing sleep problems
- Lowering the number of hospitalizations

Effective coping requires understanding the challenge, thinking through solutions, and asking for support.

CANCER REHABILITATION SERVICES

Many cancer centers and hospitals offer a variety of cancer rehabilitation services to their patients and are able to help identify local resources. Survivors and family members are encouraged to be active, informed partners in the rehabilitation process and to talk with a nurse, social worker, or patient educator about the services they are interested in. These may include:

☐ Certified health and fitness programs

These programs help survivors build strength, endurance, and mobility.

Recommendations or referral: ______________________
____
____
____
____

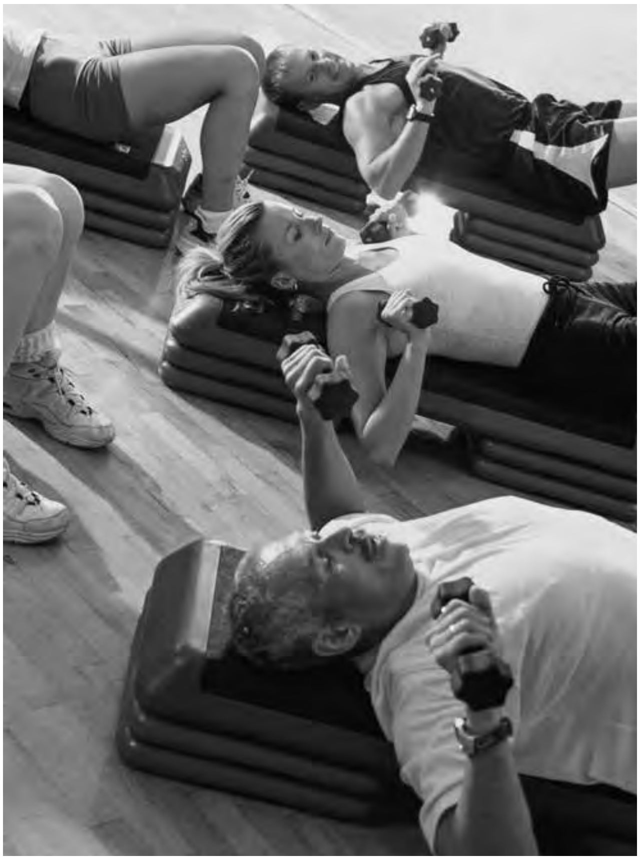

☐ Clinical trials for survivors

These research studies focus on addressing a person's quality of life after cancer treatment.

Recommendations or referral: ______________________
____
____
____
____

☐ Family counseling

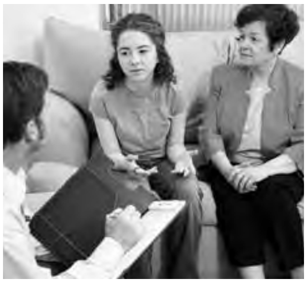

When meeting with a family, a counselor listens objectively to all participants and helps identify how specific thoughts and behaviors could be contributing to conflict. Family members learn new ways to support each other during stressful times.

Recommendations or referral: ______________________
____
____
____
____

☐ Genetic counseling

A trained genetic counselor will help you and your family understand your risk of developing an inherited medical condition (a condition passed from parent to child), such as a type of cancer, based on your personal and family medical history. The genetic counselor also explains the available genetic tests; the cancer screening, prevention, and treatment options; and serves as an ongoing resource for support.

Recommendations or referral: ______________________
____
____
____
____

☐ **Home care services**

Home care consists of a range of professional health care and supportive services delivered in the home, including help with daily activities, such as eating, drinking, dressing, bathing, using the toilet, cooking, and basic housekeeping.

Recommendations or referral: ________________________

____

____

____

____

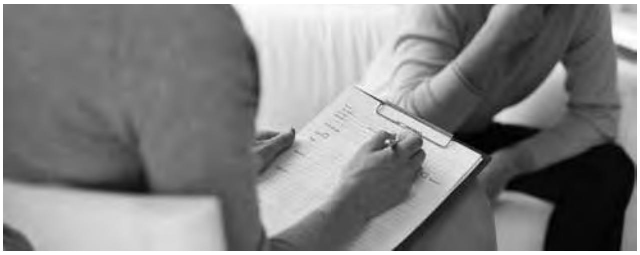

☐ **Individual counseling**

This provides one-on-one interaction with a counselor to talk about troubling circumstances, thoughts, and feelings. The counselor will listen attentively, express caring concern, ask questions, and offer feedback.

Recommendations or referral: ________________________

____

____

____

____

☐ **Marriage/couples therapy**

The goal of this type of counseling is to help couples respond to challenges and associated emotions in healthy ways. It focuses on improving relationships and resolving conflict.

Recommendations or referral: ________________________

____

____

____

____

☐ **Nutritional planning**

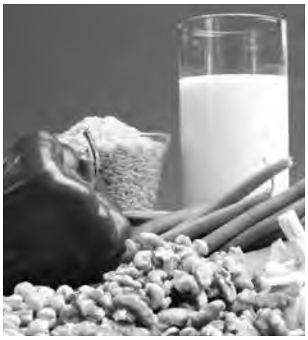

A registered dietitian can provide guidance on a survivor's specific nutritional needs, including meal planning, to regain and maintain a healthy weight and lifestyle.

Recommendations or referral: ________________________

____

____

____

____

☐ **Occupational therapy**

Occupational therapy focuses on helping people with an illness, injury, or disability develop the skills they need to accomplish daily tasks, such as self-care and work, so they can regain or maintain their independence.

Recommendations or referral: ________________________

____

____

____

____

☐ **Oncology social worker consultation**

Oncology social workers provide education, information services, discharge and home care planning services, and referrals to community resources. They also help survivors adjust to life after treatment by providing counseling on ways to cope with a variety of post-treatment issues, including practical, financial, and work-related challenges.

Recommendations or referral: ________________________

____

____

____

____

☐ **Pain management**

Nearly all cancer-related pain can be successfully treated or managed. After a thorough medical assessment, your doctor or clinician will work with you to develop a pain management plan that may include medication, physical and occupational therapy, relaxation, distraction, hypnosis, biofeedback, nutritional support, and acupuncture. Some hospitals have pain specialists to help patients and survivors with pain that is difficult to control.

Recommendations or referral: ________________________

____

____

____

____

Learn more about pain at **www.cancer.net/pain** or by reading a copy of *ASCO Answers* Managing Cancer Related Pain.

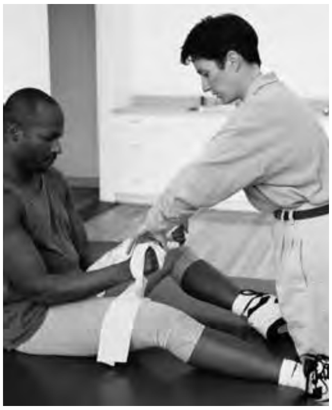

☐ **Physical therapy**

A physical therapist will evaluate any nerve, muscle, or fitness problems that make it difficult for a person to function well on a daily basis and then develop a care plan. This plan usually includes exercises and other recommendations that improve a person's physical functioning and help prevent further difficulties.

Recommendations or referral: ________________________

____

____

____

____

☐ **Recreational therapy**

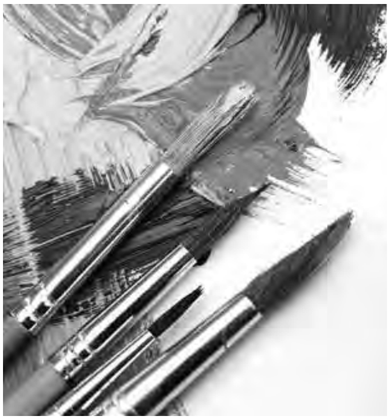

Recreational therapists work with survivors to reduce stress, anxiety, and depression through music, games, exercise, and arts and crafts.

Recommendations or referral: ________________________

____

____

____

____

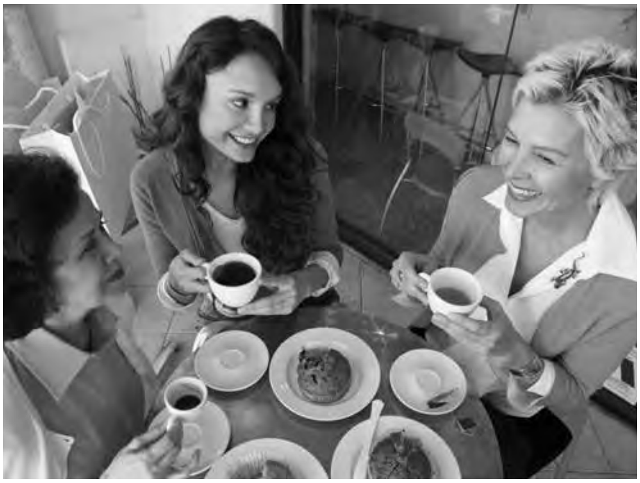

☐ **Survivor matching programs**

Also called buddy programs, these groups connect survivors with similar diagnoses, situations, or concerns to provide one-on-one peer support.

Recommendations or referral: ________________________

____

____

____

____

☐ Tobacco cessation programs

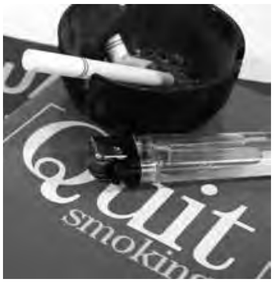

These programs provide support and resources for quitting tobacco use. A variety of treatments and resources are available for people who want to stop using tobacco, including medications and counseling. Your chances of successfully quitting are better if you follow a comprehensive plan and build a support network.

Recommendations or referral: ______________________

____

____

____

____

Learn more about stopping tobacco use by visiting **www.cancer.net/tobacco** or by reading a copy of Stopping Tobacco Use After a Cancer Diagnosis.

☐ Vocational (career) counseling

Career counselors help survivors find or keep a satisfying job, especially if their cancer experience has reshaped their career priorities and caused them to question whether they fit with their current job. Career counselors can also provide guidance on how to network effectively, teach interviewing skills, and help organize résumés by experience and skills, instead of by date.

Recommendations or referral: ______________________

____

____

____

____

For more information about support resources and links to national organizations, online support communities, and age specific resources, visit **www.cancer.net/support.**

QUESTIONS TO ASK ABOUT CANCER REHABILITATION

- Would I benefit from cancer rehabilitation services? If so, what kinds of services?
- Who can help me understand what my insurance will cover in terms of cancer rehabilitation services?
- What are other ways I can stay as healthy as possible after treatment?

NOTES:

____

____

____

____

____

____

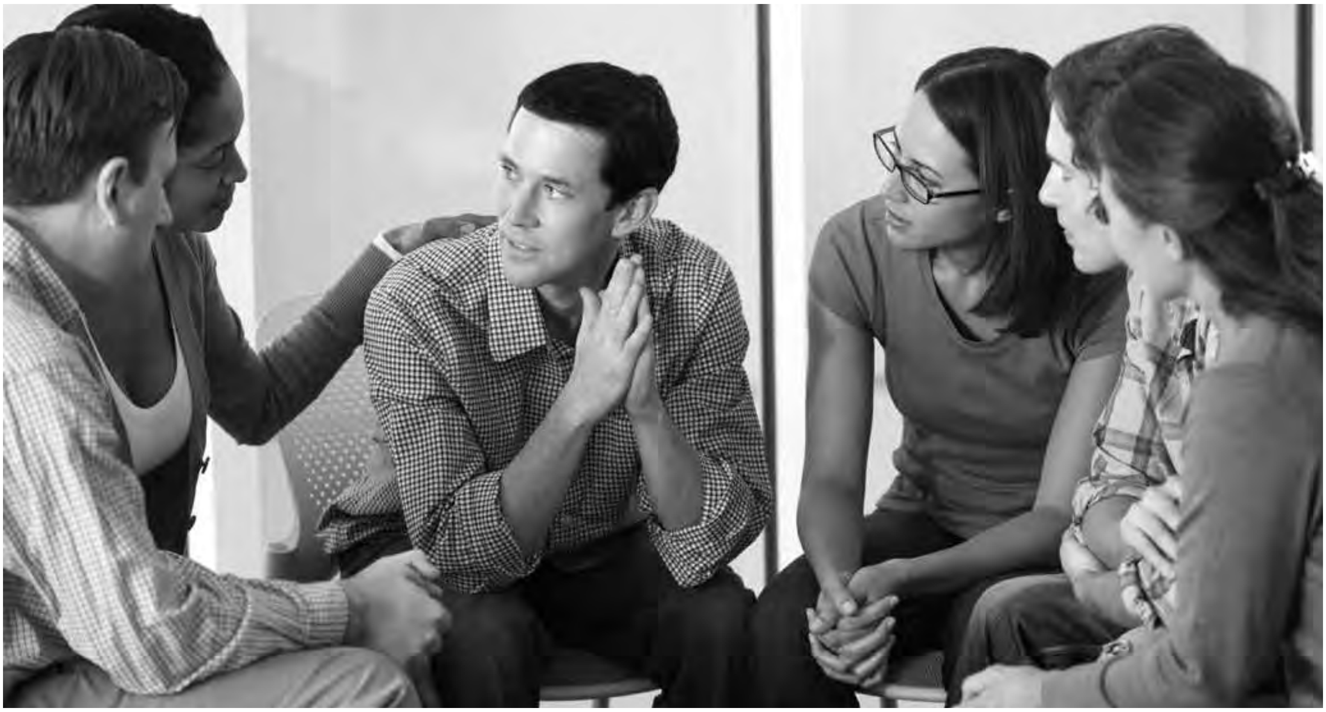

Survivorship support groups

As you move from active treatment into survivorship, know that you are not alone. You have family, friends, and community resources there to help you manage the emotional, practical, and financial issues that arise as you transition back into "normal" life. Still, some survivors find it is helpful to talk with people who know what they are going through from first-hand experience.

Support groups offer the chance for people to talk about their experiences with other survivors. Group members can share feelings and experiences that seem too strange or too difficult to share with family and friends. The group experience, which is often led by a trained counselor, social worker, or psychologist, often creates a sense of belonging that helps each person feel less alone and more understood. Sharing feelings and fears with others who understand may also help reduce stress.

In addition to sharing feelings and experiences, support group members sometimes discuss practical information, such as what to expect after treatment, how to manage pain and other long-term side effects, how to deal with the fear of recurrence, and how to communicate with health care providers and family members. These informational support groups are usually led by a professional facilitator and focus on providing cancer-related information and education. These groups often invite speakers who can provide expert information.

Finding the right support group depends on your needs and personality. Some people may need emotional support, while others may prefer an emphasis on information and education. Some people join a group that is open to all individuals with cancer, while others may prefer a group that is more specialized, such as for those with a specific type of cancer (like breast cancer or prostate cancer) or a group that is dedicated to post-treatment survivors.

In recent years, online support groups have become more popular. These groups may be a particularly good option for people who live in rural areas or for those without easy access to transportation. An online support group may allow people with rarer types of cancer to communicate with others with the same type of cancer. Online support

groups may also be a good choice for those who do not feel comfortable sharing their experiences face-to-face.

Online communities or social networking sites can connect you with other survivors who share common interests or who are in a situation similar to yours. Many cancer advocacy organizations have online support groups as well.

Other support resources

Some people with cancer may not be interested in joining a support group or may find that support groups are not helpful for them. If this is true for you, there are other ways to find support that you may want to consider, such as:

- Talking with a friend
- Individual counseling or therapy
- Asking a doctor, clinician, or nurse specific questions
- Talking with someone who is a part of your spiritual or religious community
- Visiting the learning resource center at the hospital or cancer center where you received treatment
- Focusing on other enjoyable activities

Caregivers play an important role in the care of people with cancer. Learn more about caregiving by visiting **www.cancer.net/caregiving** or by reading a copy of the *ASCO Answers* Guide to Caregiving.

QUESTIONS TO ASK ABOUT FINDING SUPPORT

- What post-treatment support services are available to me? To my family?
- Where can I find resources for a child? For a teenager? For a young adult? For an older adult?
- Does this hospital or cancer center have a learning resource center? If so, where is it located?
- Can you recommend a social worker or other professional who can help me find additional survivorship support services?

NOTES:

Making a Difference

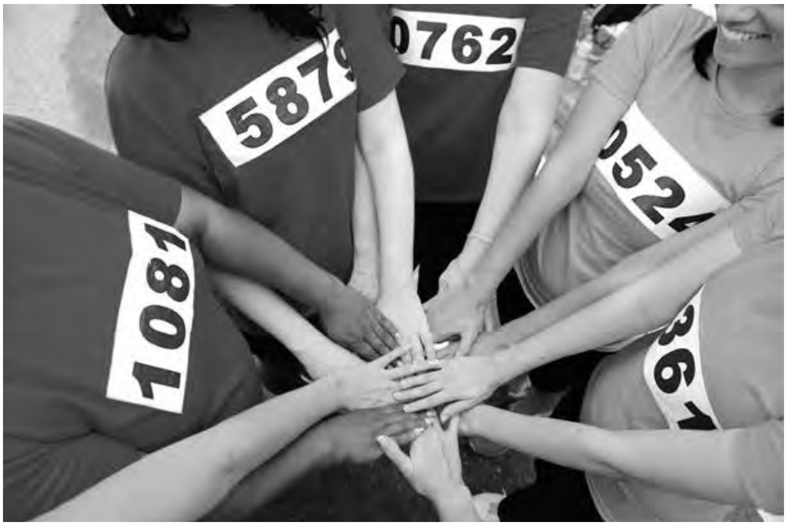

Positive feelings often arise during the transition to survivorship. Many survivors express a strong desire to "give something back" because of the care and kindness they received. Many realize they have a lot of valuable experience that can help others facing cancer. If you are interested in giving back, think about your own interests, strengths, and areas of expertise and how various organizations could use them to help further their mission.

There are a number of ways for survivors to make an important difference in someone else's life and often make a positive difference in their own life at the same time. Many survivors say sharing their time makes them feel good, helps build new friendships, and widens their network of support. These opportunities include:

Volunteering makes an important difference in someone else's life while making a positive difference in your own.

Service and support. Through these programs, survivors provide information and help people with cancer find ways to cope. Volunteer hotline counselors are trained to give easy-to-understand information over the phone and lend support by listening to patients' concerns. Some support groups are organized and run by cancer survivors. There are also cancer support programs you can get involved in that offer emotional and practical support to patients and their families by providing needed items, such as wigs, scarves, and books.

Awareness and education. Through workshops and presentations at schools, workplaces, health fairs, and on the internet, cancer organizations raise awareness about this disease and educate people about cancer prevention and screening. Many also provide tips about maintaining a healthy lifestyle and follow-up care after treatment ends. Survivors can help by teaching sessions about cancer at their workplace, community center, or place of worship; providing office services and help with event planning and logistics for local cancer organizations; or joining committees that plan new educational programs.

Fundraising. Cancer organizations usually need to raise money to maintain services and programs, such as free cancer screenings, transportation assistance and financial aid for patients and their families, outreach programs, education, and research. Survivors often participate in fundraising activities, such as races

and other sporting events, luncheons or dinners, plays or concerts, fashion shows, and auctions.

Advocacy. Being an advocate means supporting and speaking in favor of a specific cause. Survivors help lead local and even national efforts to develop or change cancer policy issues, such as access to health care and funding for cancer research. By working with cancer organizations, advocates can support legislation that helps people with cancer and their families and speak out about issues affecting people with cancer.

Survivors also advocate for cancer research by supporting clinical trials. Individual patient advocates can join a clinical trial cooperative group sponsored by the National Cancer Institute, review research grants, assist in the development of clinical trial protocols and informed consent forms, participate in community outreach and education regarding clinical research, help reduce barriers to participation in clinical research, and recruit patients to participate in specific clinical trials.

To learn more about advocacy and other ways to volunteer as a survivor, visit **www.cancer.net/advocacy.**

QUESTIONS TO ASK ABOUT VOLUNTEERING AND ADVOCACY

- How can I find cancer-related organizations to volunteer with?
- What are other ways I can support people living with cancer?
- How can I get involved in cancer research?

NOTES:

Survivorship Dictionary

Active treatment: The period when a person is having surgery, chemotherapy, radiation therapy, or other treatment to slow, stop, or eliminate the cancer.

Acute survivorship: A term describing the period when a person is diagnosed with cancer and/or receiving active treatment.

Americans with Disabilities Act (ADA): A federal (national) law that protects people with disabilities from discrimination. It requires employers to make reasonable accommodations in the workplace for qualified individuals with a disability. Learn more at www.ada.gov.

Anxiety: Feelings of nervousness, fear, apprehension, and worry.

Case manager: A health care professional who helps coordinate a person's medical care before, during, and after treatment. At a medical center, a case manager may provide a wide range of services, including managing treatment plans, coordinating health insurance approvals, and locating support services. Insurance companies also employ case managers.

Chemotherapy: The use of drugs to destroy cancer cells.

Clinical trial: A research study that involves volunteers. Many clinical trials test new approaches to treatment and/or prevention to find out whether they are safe, effective, and possibly better than the treatment doctors currently use.

Depression: Defined as having a low mood and/or feeling numb consistently for more than two weeks, every day and much of the day.

Extended survivorship: A term describing the period when a person has just completed active treatment, usually measured in months.

Fertility: Ability for women to become pregnant or have children. For men, fertility refers to the ability to provide healthy sperm.

Follow-up care plan: A personalized schedule of follow-up examinations and tests that a doctor recommends after the active treatment period. This may include regular physical examinations and/or medical tests to monitor the person's recovery for the coming months and years. This may also be called a survivorship care plan; it is often used in conjunction with a treatment summary.

Hormonal therapy: Treatment that removes or blocks hormones to destroy or slow the growth of cancer cells. Also called endocrine therapy.

Imaging test: A procedure that creates pictures of internal body parts, tissues, or organs to make a diagnosis, plan treatment, check whether treatment is working, observe a disease over time, or check for a recurrence.

Laboratory test: A procedure that evaluates a sample of blood, urine, or other substance from the body to make a diagnosis, guide treatment, check whether treatment is working, observe a disease over time, or check for a recurrence.

Late effects: Side effects of cancer or its treatment that occur months or years after the active treatment period has ended.

Learning resource center: A location in a hospital or cancer center where patients and families can get information about health-related topics and learn about support resources. Also called a health or hospital library.

Long-term side effects: Side effects that linger after cancer treatment has ended.

Metastasis: Cancer that has spread to other parts of the body from the place where it started.

Oncologist: A doctor who specializes in treating cancer. The main types include medical, surgical, radiation, gynecologic, and pediatric oncologists.

Oncology nurse: A nurse who specializes in caring for people with cancer.

Palliative care: Any form of treatment that concentrates on reducing a person's symptoms or treatment-related side effects, improving quality of life, and supporting patients and their families. Also called supportive care.

Patient navigator: A person, often a nurse or social worker, who helps guide survivors, families, and caregivers through the health care system by offering services such as arranging financial support, coordinating care among several doctors, and providing emotional support.

Patient Protection and Affordable Care Act: Often called "health care reform," this is a 2010 federal law that changed certain rules regarding health insurance coverage in the United States. Learn more at www.HealthCare.gov.

Physiatrist: A medical doctor who treats injuries and illnesses that affect how a person moves, including the treatment of pain. Also called a rehabilitation specialist.

Prognosis: Chance of recovery; a prediction of the outcome of a disease.

Primary cancer: The original (first) cancer with which a person was diagnosed. The primary site is where the cancer began.

Psychiatrist: A medical doctor who has special training in preventing, diagnosing, and treating mental, emotional, and behavioral problems.

Psychologist: A specialist who can talk with patients and their families about emotional and personal matters and can help them make decisions.

Quality of life: An overall sense of well-being and satisfaction with life.

Radiation therapy: The use of high-energy x-rays or other particles to destroy cancer cells. Also called radiotherapy.

Recurrence: Cancer that has returned after a period during which the cancer could not be detected. Local recurrence means the cancer has come back in the same general area where the original cancer was located. Regional recurrence refers to cancer that has come back in the lymph nodes or other tissues near the original cancer site. Distant recurrence refers to cancer that has come back and has spread to other parts of the body, usually by traveling through the lymphatic system or bloodstream.

Referral: Recommendation provided by a doctor to get help or information from another health care professional, specialist, or resource. Insurance companies often require a referral before they will cover visits to other health care professionals or specialists.

Rehabilitation: Services and resources that help a person with cancer obtain the best physical, social, psychological, and work-related functioning during and after cancer treatment. The goal of rehabilitation is to help people regain control over many aspects of their lives and remain as independent and productive as possible.

Remission: Period in which the signs and symptoms of cancer have disappeared. This can be temporary or permanent. Also called "no evidence of disease" or NED.

Risk: The likelihood of an event.

Secondary cancer: A new primary cancer (a different type of cancer) that develops after treatment for the first type of cancer.

Side effect: An undesirable result of treatment, such as fatigue, diarrhea, or sexual problems.

Social worker: A professional who helps people cope with everyday tasks and challenges before, during, and after treatment. Social workers may work for a hospital, a service agency, or a local government and help address financial problems, explain insurance benefits, provide access to counseling, and more.

Stage: A way of describing where the cancer is located, if or where it has spread, and whether it is affecting other parts of the body.

Surgery: The removal of cancerous tissue from the body during an operation.

Survivorship: This term means different things to different people. Common definitions include having no disease after the completion of treatment and the process of living with, through, and beyond cancer.

Targeted treatment: Treatment that targets specific genes, proteins, or other molecules that contribute to cancer growth and survival.

Treatment summary: A written summary of the therapy(ies) that a person had during the active treatment period. This is often used in conjunction with a follow-up care plan to help monitor a survivor's long-term health.

For more definitions of common cancer related terms, visit **www.cancer.net/cancerterms**.

Looking for Other Patient Information Resources?

Cancer.Net offers a variety of guides, booklets, and fact sheets to help patients learn more about their disease and treatment.

ASCO ANSWERS GUIDES

ASCO Answers Guides feature comprehensive information about the diagnosis, treatment, side effects, and psychosocial effects of a specific cancer type, as well as practical information for patients and families. Topics include:

- Breast Cancer
- Colorectal Cancer
- Non-Small Cell Lung Cancer
- Small Cell Lung Cancer
- Prostate Cancer
- Survivorship
- Caregiving

ASCO ANSWERS FACT SHEETS

ASCO Answers Fact Sheets provide a one-page (front and back) introduction to a specific type of cancer or cancer-related topic. Each includes an overview, illustration, words to know, and questions to ask the health care team. Cancer.Net has more than 65 fact sheets available (including some in Spanish), covering different cancer types, diagnosis and treatment, and side effects.

ASCO ANSWERS BOOKLETS

ASCO Answers Booklets provide in-depth, practical guidance on specific topics in cancer care. Learn about:

- Advanced Cancer Care Planning
- Managing Cancer-Related Pain
- Managing the Cost of Cancer Care
- Managing Your Weight After a Cancer Diagnosis
- Palliative Care
- Stopping Tobacco Use After a Cancer Diagnosis

For Patients and Caregivers: If you are interested in additional educational materials, visit www.cancer.net/ascoanswers to find all of our available materials in electronic format.

For Oncology Professionals: Bulk quantities of high-quality print materials are available for purchase. Visit www.cancer.net/estore or call 1-888-273-3508 to place your order. To request free promotional materials for your practice, please send an email to contactus@cancer.net.

Cancer.Net

Doctor-Approved Patient Information from ASCO®

WE WANT TO HEAR FROM YOU

If you found this material helpful or if you have comments or suggestions about how they could be better, please let us know at contactus@cancer.net.

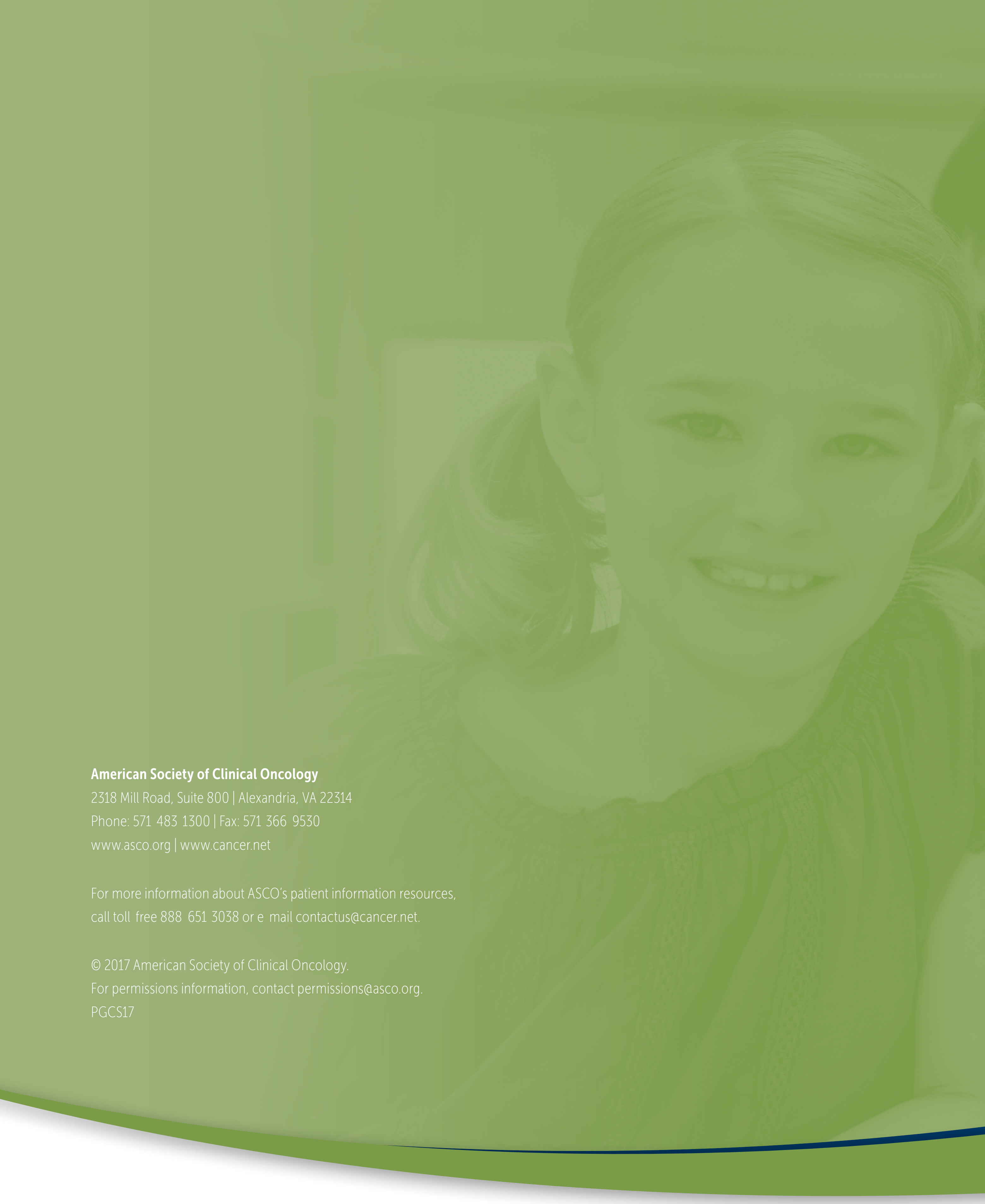

American Society of Clinical Oncology
2318 Mill Road, Suite 800 | Alexandria, VA 22314
Phone: 571 483 1300 | Fax: 571 366 9530
www.asco.org | www.cancer.net

For more information about ASCO's patient information resources, call toll free 888 651 3038 or e mail contactus@cancer.net.

© 2017 American Society of Clinical Oncology.
For permissions information, contact permissions@asco.org.
PGCS17
