# Supplementary material for: A pilot randomized controlled trial of an online intervention for Hodgkin lymphoma survivors to increase knowledge about late effects and recommended screening
Source: J Cancer Surviv. 2024 Apr 20;19(6):1781–92. doi: 10.1007/s11764-024-01587-2 (PMC11490585; doi:10.1007/s11764-024-01587-2)
Supplement: Supplementary file 2 — Supplementary file2 (PDF 121 KB) [file 11764_2024_1587_MOESM2_ESM.pdf]

UNIVERSITY OF MINNESOTA

Twin Cities Campus

Division of Hematology, Oncology and Transplantation
Department of Medicine
Medical School

MMC 480
420 Delaware Street S.E.
Minneapolis, MN 55455
612-624-0123
Fax: 612-625-6919

MAPS Side Effects Info Sheet

People who received treatment for Hodgkin lymphoma have an increased risk of developing other diseases or conditions later in life. This is because both chemotherapy and radiation therapy can cause permanent damage. Treatments have improved in the last 30 years, and now patients are less likely to experience late effects; however, there is still some risk. Therefore, it is important that patients see their doctors regularly for follow-up care and watch for any new side effects.

- **Infertility.** Radiation therapy to the pelvic area can cause infertility unless the ovaries or testicles are shielded during treatment. Also, teenagers and adults who receive chemotherapy may be at higher risk for low sperm counts (for men) or damage to the ovaries (for women). The risk of infertility is low after ABVD chemotherapy for Hodgkin lymphoma but is much higher after BEACOPP. Stem cell transplantation usually causes infertility in men. It is unusual, but not impossible, for women to become pregnant after a stem cell transplant.
  Patients who are considering having a family should discuss fertility preservation with their doctor before starting treatment. Learn more about fertility concerns and preservation for men and women.
- **Second cancers.** Some survivors of Hodgkin lymphoma have a higher risk of developing a secondary cancer, especially acute myeloid leukemia (following certain types of chemotherapy or radiation therapy) non-Hodgkin lymphoma, lung cancer, or breast cancer. The risk of a secondary cancer is likely to decrease in the future because the treatments used now have fewer risks. Patients can lower their risk of developing a secondary cancer by limiting or avoiding other risk factors, such as smoking. It is also important that women who receive radiation therapy to the chest begin regular breast cancer screening at age 40 or 8 years after treatment, whichever comes first.
- **Lung and heart damage.** Patients who received anthracyclines (doxorubicin) or bleomycin during chemotherapy have a higher risk of both heart and lung damage. Radiation therapy to the chest area also can cause lung damage and increase the risk of heart disease. It is very important that men and women who receive radiation therapy to the chest limit other risk factors that may lead to heart damage by not smoking, getting regular exercise, by

monitoring and maintaining healthy blood pressure and cholesterol levels, and by making healthy food choices.

- **Thyroid problems.** Radiation therapy to the neck area can cause problems with the thyroid gland, most commonly hypothyroidism. Hypothyroidism is when the body produces too little thyroid hormone, which regulates metabolism. This problem can be checked by using a blood test and managed by taking a thyroid hormone supplement pill.
- **Emotional issues.** Survivors of Hodgkin lymphoma have a higher risk of depression and other emotional concerns.
  - Care for people diagnosed with lymphoma doesn't end when active treatment has finished. Your health care team will continue to check to make sure the cancer has not returned, manage any side effects and late effects of treatment, and monitor your overall health. This is called follow-up care.
  - Your follow-up care may include regular physical examinations, medical tests, or both. Doctors want to keep track of your recovery in the months and years ahead. How often a person needs follow-up care and which tests are performed depend on several factors, including the original extent of the Hodgkin lymphoma and the type of treatment. Tests like CT scans and PET-CT scans should be performed after treatment ends to make sure that the disease is in complete remission. However, research has shown that routine subsequent scans, sometimes called "surveillance imaging," are not usually necessary but should be considered if there are any signs or symptoms of the lymphoma coming back. Talk with your doctor whether tests such as CT scans and PET-CT scans should be repeated, as well as how often you should have physical examinations.
  - Patients who have had Hodgkin lymphoma should get a flu shot every year. It may be recommended that some survivors get an immunization against pneumonia, which may be done every 5 to 7 years.
  - Learn more about the importance of follow-up care.

Watching for recurrence

One goal of follow-up care is to check for a recurrence. Cancer recurs because small areas of cancer cells may remain undetected in the body. Over time, these cells may increase in number until they show up on test results or cause signs or symptoms. During follow-up care, a doctor familiar with your medical history can give you personalized information about your risk of recurrence. Your doctor will also ask specific questions about your health.

In general, each follow-up visit includes a discussion with the doctor, a physical examination, and blood tests. During some visits, scans may be done. At most cancer centers, follow-up visits are scheduled every 2 to 3 months for the first 1 to 2 years after treatment is completed, which is when the risk of recurrence is highest. After that, the time between visits increases over time.

Later visits may only be 2 to 3 times per year until 5 years have passed. Then annual visits should be continued with an oncologist.

The anticipation before having a follow-up test or waiting for test results can add stress to you or a family member. This is sometimes called “scan-xiety.” Learn more about how to cope with this type of stress.

Managing long-term and late side effects

Most people expect to experience side effects when receiving treatment. However, it is often surprising to survivors that some side effects may linger beyond the treatment period. These are called long-term side effects. Other side effects called late effects may develop months or even years afterwards. Long-term and late effects can include both physical and emotional changes.

Talk with your doctor about your risk of developing such side effects based on the type of cancer, your individual treatment plan, and your overall health. If you had a treatment known to cause specific late effects, you may have certain physical examinations, scans, or blood tests to help find and manage them. Special attention should be paid to past cancer screening and detection, as well as heart risk factors, throughout the person’s lifetime. For patients who received radiation therapy to the neck or chest, monitoring thyroid gland function is important.

There is a risk of late effects that affect the heart, so annual blood pressure checks, cholesterol measurements, and management of any risk factors for heart problems may be recommended. Echocardiogram of the heart may be recommended every 5 to 10 years, especially in patients who received radiation therapy to the chest as part of their Hodgkin lymphoma treatment plan.

Follow-up care should also address the person’s quality of life, including emotional concerns. In particular, Hodgkin lymphoma survivors are encouraged to be aware of the symptoms of depression and to talk with their doctor immediately if they have such symptoms.

Keeping personal health records

You and your doctor should work together to develop a personalized follow-up care plan. Be sure to discuss any concerns you have about your future physical or emotional health. ASCO offers forms to help create a treatment summary to keep track of the cancer treatment you received and develop a survivorship care plan when treatment is completed.

This is also a good time to decide who will lead your follow-up care. Some survivors continue to see their oncologist, while others transition back to the care of their family doctor or another health care professional. This decision depends on several factors. Including the type and stage of cancer, side effects, health insurance rules, and your personal preferences.

If a doctor who was not directly involved in your cancer care will lead your follow-up care, be sure to share your cancer treatment summary and survivorship care plan forms with him or her and will all future health care providers. Details about your cancer treatment are very valuable to the health care professionals who will care for you throughout your lifetime.

What is survivorship?

The word “survivorship” means different things to different people. Common definitions include:

- Having no signs of cancer after finishing treatment.
- Living with, through, and beyond cancer. According to this definition, cancer survivorship begins at diagnosis and includes people who continue to have treatment over the long term, either to reduce the risk of recurrence or to manage chronic disease.

Survivorship is one of the most complicated parts of having cancer. This is because it is different for everyone.

Survivors may experience a mixture of strong feelings, including joy, concern, relief, guilt, and fear. Some people say they appreciate life more after a cancer diagnosis and have gained a greater acceptance of themselves. Others become very anxious about their health and uncertain about coping with everyday life.

Survivors may feel some stress when their frequent visits to the health care team end after completing treatment. Often, relationships built with the cancer care team provide a sense of security during treatment, and people miss this source of support. This may be especially true when new worries and challenges surface over time, such as any late effects of treatment, emotional challenges including fear of recurrence, sexuality and fertility concerns, and financial and workplace issues.

Every survivor has individual concerns and challenges. With any challenge, a good first step is being able to recognize your fears and talk about them. Effective coping requires:

- Understanding the challenge you are facing
- Thinking through solutions
- Asking for and allowing the support of others
- Feeling comfortable with the course of action you choose

Many survivors find it helpful to join an in-person support group or an online community of survivors. This allows you to talk with people who have had similar firsthand experiences. Other options for finding support include talking with a friend of member of your health care team, individual counseling, or asking for assistance at the learning resource center of the place where you received treatment.

Changing the role of caregivers

Family members and friends may also go through periods of transition. A caregiver plays a very important role in supporting a person diagnosed with cancer, providing physical, emotional, and practical care on a daily or as-needed basis. Many caregivers become focused on providing this support, especially if the treatment period lasts for many months or longer.

However, as treatment is completed, the caregiver's role often changes. Eventually, the need for caregiving related to the cancer diagnosis will become much less or end. Caregivers can learn more about adjusting to life after caregiving in this article.

A new perspective on your health

For many people, survivorship serves as a strong motivator to make positive lifestyle changes.

People recovering from Hodgkin lymphoma are encouraged to follow established guidelines for good health, such as not smoking, limiting alcohol, eating well, and managing stress. Regular physical activity can help rebuild your strength and energy level. Your health care team can help you create an appropriate exercise plan based upon your needs physical abilities, and fitness level. Learn more about making healthy lifestyle choices.

It is important to have recommended medical checkups and tests (see Follow-up Care) to take care of your health. Cancer rehabilitation may also be recommended, and this could mean any of a wide range of services such as physical therapy, career counseling, pain management, nutritional planning, and/or emotional counseling. The goal of rehabilitation is to help people regain control over many aspects of their lives and remain as independent and productive as possible. Talk with your doctor to develop a survivorship care plan that is best for your needs.

Looking for more Survivorship resources?

For more information about cancer survivorship, explore these related items. Please note that these links will take you to other sections of Cancer.net:

- **ASCO Answers Cancer Survivorship Guide:** Get this 44-page booklet that helps people transition into life after treatment. It includes blank treatment summary and survivorship care plan forms. The booklet is available as a PDF, so it is easy to print out.
- **Cancer.Net Patient Education Video:** View a short video lead by an ASCO expert that provides information about what comes next after finishing treatment.
- **Survivorship Resources:** Cancer.Net offers an entire area of this website with resources to help survivors, including those in different age groups.

Adapted from Cancer.Net
